# Structure of UBE2K–Ub/E3/polyUb reveals mechanisms of K48-linked Ub chain extension

**DOI:** 10.1038/s41589-021-00952-x

**Published:** 2022-01-13

**Authors:** Mark A. Nakasone, Karolina A. Majorek, Mads Gabrielsen, Gary J. Sibbet, Brian O. Smith, Danny T. Huang

**Affiliations:** 1 Cancer Research UK Beatson Institute, Glasgow, UK; 2 MVLS Structural Biology and Biophysical Characterisation Facility, University of Glasgow, Glasgow, UK; 3 Institute of Molecular Cell and System Biology, University of Glasgow, Glasgow, UK; 4 Institute of Cancer Sciences, University of Glasgow, Glasgow, UK

**Keywords:** X-ray crystallography, Enzyme mechanisms, Post-translational modifications, NMR spectroscopy

## Abstract

Ubiquitin (Ub) chain types govern distinct biological processes. K48-linked polyUb chains target substrates for proteasomal degradation, but the mechanism of Ub chain synthesis remains elusive due to the transient nature of Ub handover. Here, we present the structure of a chemically trapped complex of the E2 UBE2K covalently linked to donor Ub and acceptor K48-linked di-Ub, primed for K48-linked Ub chain synthesis by a RING E3. The structure reveals the basis for acceptor Ub recognition by UBE2K active site residues and the C-terminal Ub-associated (UBA) domain, to impart K48-linked Ub specificity and catalysis. Furthermore, the structure unveils multiple Ub-binding surfaces on the UBA domain that allow distinct binding modes for K48- and K63-linked Ub chains. This multivalent Ub-binding feature serves to recruit UBE2K to ubiquitinated substrates to overcome weak acceptor Ub affinity and thereby promote chain elongation. These findings elucidate the mechanism of processive K48-linked polyUb chain formation by UBE2K.

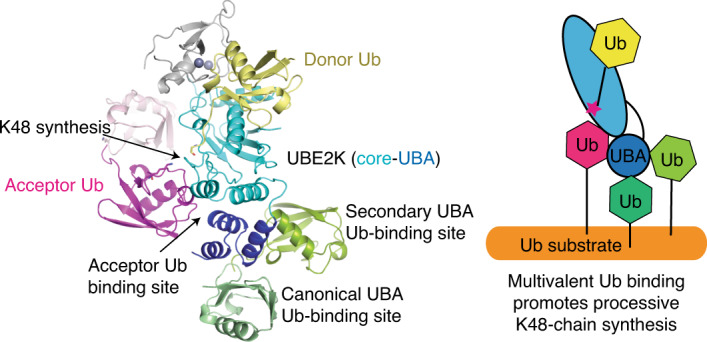

## Abstract (web summary)

K48-linked ubiquitin chains direct substrates for proteasomal degradation. Nakasone et al. provide the molecular basis for K48-linked ubiquitin chain synthesis by UBE2K and reveal a multivalent ubiquitin-binding mechanism of UBA in extending the ubiquitin chain.

## Main

Across eukaryotic life ubiquitin is an adaptable post-translational modification vital for numerous signaling pathways^1^. While in most cases only the Ub ligases (E3s) select substrates for Ub modification^2^, for RING E3s, the ubiquitin-conjugating enzymes (E2s) govern how polyUb chains are constructed by defining the internal Ub–Ub linkages^3,4^. E2 selection for substrate ubiquitination is typically initiated by E3s, which catalyze Ub attachment through recruitment of E2 thioesterified Ub (E2~Ub) and substrate^5^. RING E3s can utilize two different E2s depending on the task: one for priming a substrate with Ub and a second to subsequently extend the primed Ub with a distinct polyUb signal^6–9^. These functional differences divide E2s into two mechanistically distinct classes, priming and chain elongating^10,11^. Although RING E3s stimulate both types of E2s, the presence of a Ub acceptor (Ub^A^) binding site is what distinguishes between priming and elongating. In general the acceptor site of elongating E2s is specially adapted for Ub and often accompanied by factors outside the ubiquitin-conjugating (UBC) domain or other binding partners to facilitate elongation^5^.

Formation of specific internal Ub–Ub linkages is critical for downstream signaling^12,13^. Coupled proteasomal targeting and degradation is primarily initiated by K48-linked polyUb attached to a substrate^14^. Out of the ~35 human E2s only UBE2K and the UBE2R family are reported to exclusively form K48-linked unanchored polyUb^5,7,15,16^. The exact functions of UBE2K are poorly understood and supposedly more diverse than the UBE2R family, as UBE2K is reported to interact with many more RING E3 Ub ligases^17–20^. The orientation of Ub^A^ is perhaps more important for understanding linkage specificity, but the transient nature of this interaction has proven difficult to unlock^21^.

In this study we elucidate how UBE2K functions with RING E3s and K48-linked di-Ub as the acceptor. Combining several chemical biology approaches has enabled us to determine the structure of a RING E3, RNF38, with a donor Ub (Ub^D^) loaded on UBE2K (UBE2K–Ub^D^) and K48-linked di-Ub in the acceptor site. This methodology is eminently transferable to other E2/E3 systems to allow such complexes to be trapped for structural studies. In this 2.4-Å resolution crystal structure, RNF38 primes UBE2K–Ub^D^, while the distal Ub of K48-linked di-Ub is accommodated between the UBA and UBC domains of UBE2K. This catalytic arrangement reveals a unique structural snapshot of K48-linked polyUb chain synthesis. We also elucidate a previously unknown role of the UBA domain in binding of diverse polyUb, thereby illustrating how UBE2K efficiently catalyzes polyubiquitination of substrates.

## Results

### RING E3s stimulate UBE2K

**Fig. 1:**
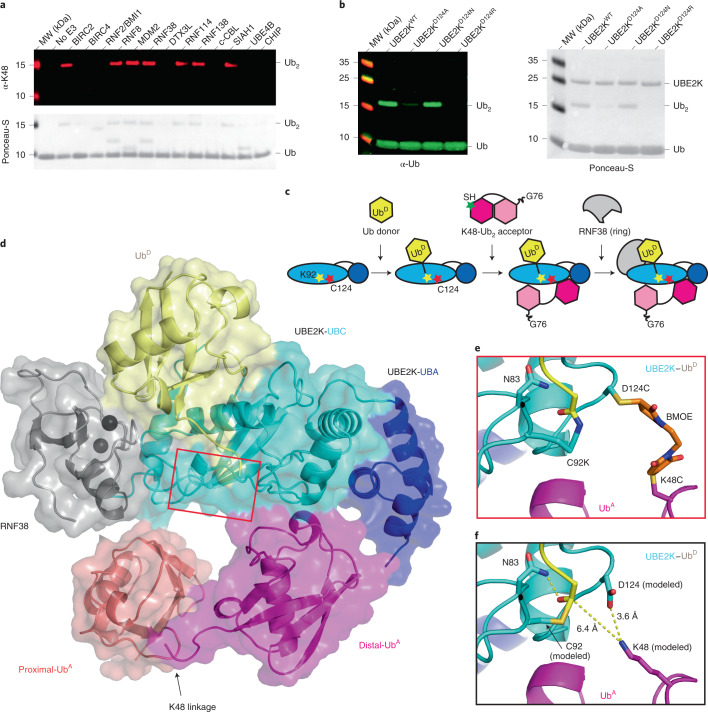
Trapping of UBE2K in complex with RING, Ub^D^ and polyUb Ub^A^. **a**, Formation of K48-linked polyUb by UBE2K is detected across a panel of E3s with anti-K48-linked Ub (top) and Ponceau-S (bottom). **b**, Di-Ub formation is probed with anti-Ub (left) and Ponceau-S (right) at a single time point for UBE2K D124 variants. **c**, The complex is created stepwise by loading Ub^D^ to C92K of UBE2K, chemical cross-linking K48C of distal Ub in K48-Ub_2_ to D124C of UBE2K and, finally, addition of the RNF38 RING domain. **d**, Structure of RNF38 RING (gray) with UBE2K (UBC cyan and UBA blue) conjugated to Ub^D^ (yellow), K48-Ub_2_ acceptor with distal K48C Ub (magenta) and proximal Ub (salmon). **e**, Close-up view of the UBE2K active site (red inset in **c**). BMOE linkage between UBE2K C124 and Ub^A^ C48 with UBE2K–Ub^D^ linkage to K92 are shown as sticks. **f**, Energy minimized model shows K48 from Ub^A^ poised toward UBE2K’s active site C92–Ub^D^ thioester bond and D124 and the C92–Ub^D^ linkage is stabilized by N83. Source data

**Extended Data Fig. 1:**
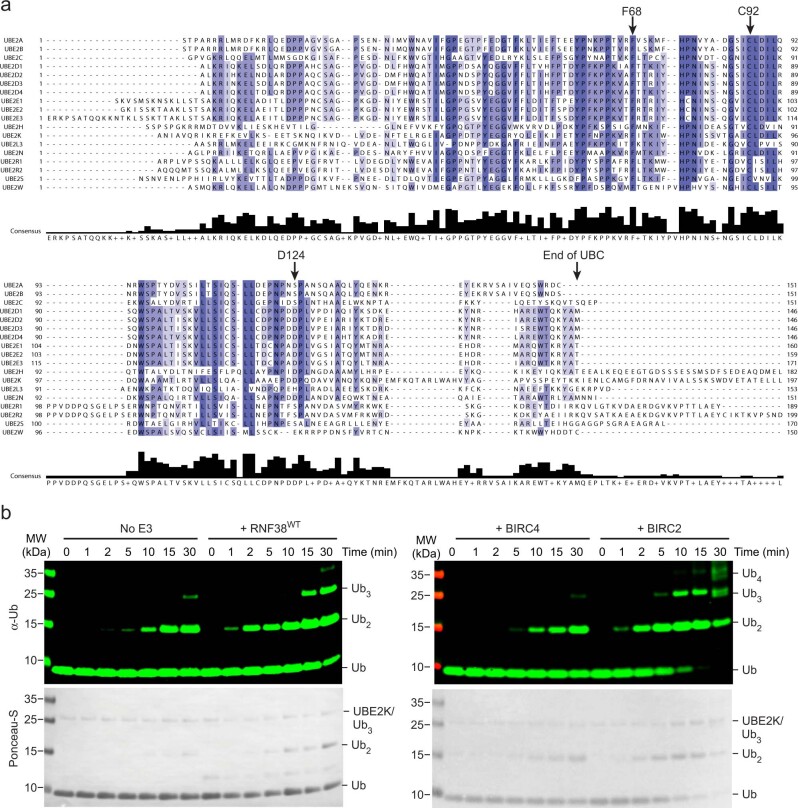
UBE2K domain and activity with RING E3s. (**a**) Sequence alignment of human E2’s reported to function with RING E3s. (**b**) Continuous turnover time course showing reduced SDS-PAGE of UBE2K^WT^-catalyzed free polyUb chains in the absence and presence of the indicated RING E3 detected with αUb (top) and ponceau-S (bottom). Source data

UBE2K is the only E2 that incorporates a UBA domain, a feature conserved across yeast (Ubc1) and human homologs (Supplementary Fig. 1). The N-terminal region of UBE2K retains conserved residues found in E2s adapted to function with RING E3s, as highlighted by the UBE2D family (Extended Data Fig. 1a). To date, RING E3 binding and stimulation of UBE2K has been sparsely reported through limited functional assays, leaving the detailed mechanism as yet undetermined. This led us to explore which RING E3s function with UBE2K. Initially a panel of RING and U-box E3 ligases were reacted with UBE2K and wild-type Ub (Ub^WT^) in a continuous turnover assay (Fig. 1a and Extended Data Fig. 1b). Unlike the UBE2D family E2s, UBE2K primarily forms unanchored polyUb in the absence of substrate^5,8,16^. We therefore assessed the reaction products for the presence of K48-linked polyUb to examine whether the expected product was formed. Interestingly, not all RING E3s stimulated UBE2K and several reported to function with the UBE2D family produced K48-linked di-Ub with UBE2K. Comparing polyUb production by UBE2K in the absence or presence of RNF38 and BIRC2 demonstrated that formation of tri-Ub was also enhanced (Extended Data Fig. 1b). Taken together these assays suggest that several RING E3s function with UBE2K to stimulate transfer of Ub^D^ to acceptor Ub.

### Design of a trapped mimic of K48-Ub_2_−UBE2K~Ub/RNF38 complex

To explain how UBE2K~Ub simultaneously coordinates Ub at the acceptor site and is stimulated by RING E3s, we designed and assembled a covalently trapped mimic of the catalytic complex. No experimentally determined structures to date represent Ub in a catalytically competent state when bound to UBE2K (Supplementary Fig. 2). In all available UBE2K–Ub complexes, regardless of the methodology used to form the complex, Ub preferentially occupies the canonical Ub-binding site on the UBA domain^17,22,23^. At best, this places K48 of Ub ~30 Å from UBE2K’s active site Cys and does not reveal how UBE2K positions acceptor Ub for catalysis. In the only available structure of UBE2K in complex with K48-Ub_2_, K48 of the distal acceptor Ub is over 35 Å from the catalytic C92 of UBE2K^22^. Predictions from molecular docking and mutagenesis analysis provide the only hint of how Ub is accommodated in UBE2K’s acceptor site^17^. Thus it remains an open question as to how UBE2K initiates and extends K48-linked Ub chains.

**Extended Data Fig. 2:**
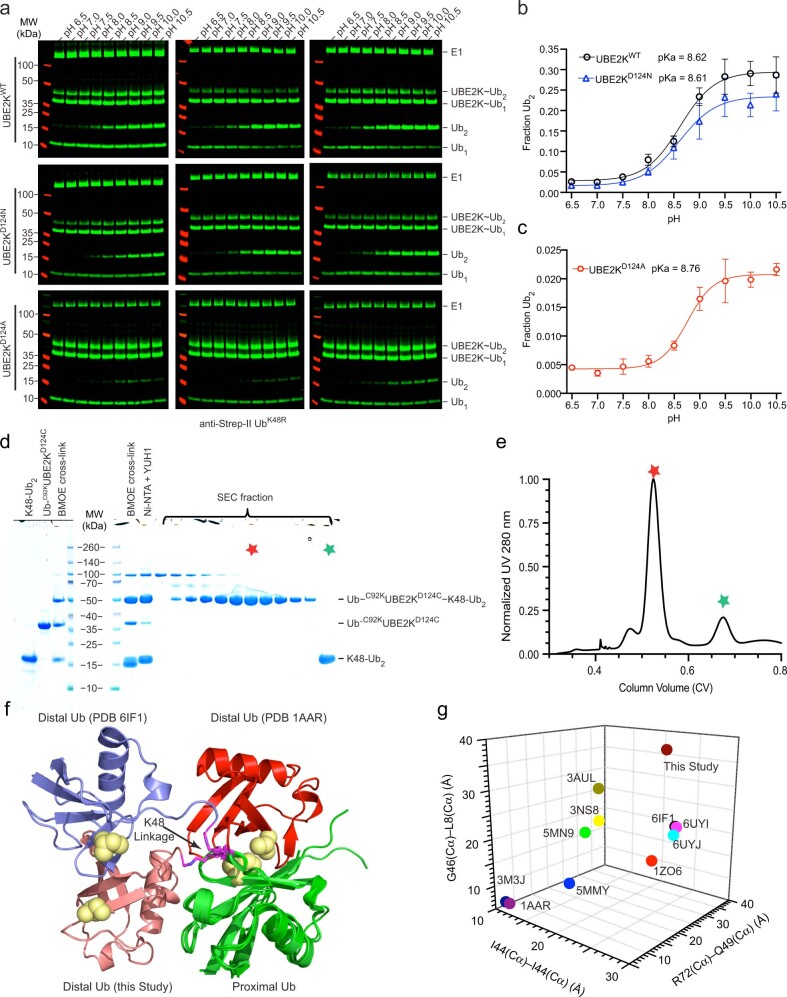
Generation and analysis of Ub–^C92K^UBE2K^D124C−K48C^Ub−^48^Ub complex. (**a**) Western blots showing di-Ub formation catalyzed by UBE2K^WT^, UBE2K^D124N^, and UBE2K^D124A^ with Strep-tag II Ub^K48R^ across the indicated pH detected with anti-Strep-tag II and performed in triplicate. (**b**) Fraction of di-Ub formed versus pH catalyzed by UBE2K^WT^ and UBE2K^D124N^ from **a**. UBE2K^D124N^ had reduced activity at higher pH compared to UBE2K^WT^. (**c**) Fraction of di-Ub formed versus pH catalyzed by UBE2K^D124A^ from **a**. The best fit of the inflection point representing the pKa for **b** and **c** are determined from (equation 1, methods), error bars are presented as ± SD from n = 3 independent experiments for each UBE2K variant. (**d**) Coomassie gel showing purification of Ub–^C92K^UBE2K^D124C−K48C^Ub−^48^Ub complex for co-crystallization with RING E3s. (**e**) Superdex 75 gel filtration profile of Ub–^C92K^UBE2K^D124C−K48C^Ub−^48^Ub complex. Red star indicates the complex as in **d**. (**f**) Structural alignment of proximal Ub (green) of K48-Ub_2_ in our structure (salmon) and PDB 1AAR (red) and 6IF1 (purple). PDB 1AAR represents the closed conformation of K48-Ub_2_, whereas K48-Ub_2_ in our structure and PDB 6IF1 adopt their own distinct open conformations. (**g**) Cα- Cα distance (Å) for indicated residues in distal and proximal Ub from structures of K48-Ub_2_.

We reasoned that the questions pertaining to stimulation by RING E3 and accommodation of Ub as the acceptor could be combined and addressed within a single complex. This led us to load a Ub^D^ via an isopeptide bond to the active site C92K of UBE2K^C92K^. UBE2K D124 juxtaposes the active site C92 and the corresponding Asp in UBE2I and UBE2N was shown to coordinate acceptor lysine and facilitate its deprotonation by lysine p*K* suppression^24,25^. We performed di-Ub formation at different pH values and found no shift in the p*K*_a_ between UBE2K^WT^ and UBE2K^D124^ variants, but UBE2K^D124A^ and UBE2K^D124R^ hindered di-Ub formation, and UBE2K^D124N^ exhibited reduced activity at high pH (Fig. 1b and Extended Data Fig. 2a–c). We posited that D124 has a minimal role in lysine p*K* suppression, but likely contributes important polar interactions with the acceptor lysine. Therefore D124 was selected as the acceptor cross-linking site and we chemically cross-linked D124C of Ub–^C92K^UBE2K^D124C^ to ^K48C^Ub via maleimide chemistry. However, this complex did not yield protein crystals with our RING E3 panel and therefore we cross-linked ^K48C^Ub−^48^Ub to Ub–^C92K^UBE2K^D124C^ (Fig. 1c and Extended Data Fig. 2d,e) to increase the likelihood for crystallization. K97R was included to prevent nonproductive UBE2K–Ub formation. Following the convention of an established nomenclature system^26^, we designate this complex Ub–^C92K^UBE2K^D124C−K48C^Ub−^48^Ub.

### Structure of the K48-Ub_2_-UBE2K–Ub/RNF38 complex

Several RING E3 domains that were active in our RING panel survey were combined with this UBE2K complex for cocrystallization, but crystals were only obtained with RNF38. The Ub–^C92K^UBE2K^D124C−K48C^Ub−^48^Ub/RNF38 crystals resulted in a 2.4-Å resolution structure detailed in Fig. 1d and Supplementary Table 1. In the structure, Ub^D^ is primed for transfer by RNF38, while the active site region of UBE2K encloses the distal Ub of K48-Ub_2_ (hereafter referred to as Ub^A^), which presents as an ‘open’ conformation (Extended Data Fig. 2f,g). The UBE2K^D124C-BMOE-K48C^Ub linkage juxtaposes the Ub–^C92K^UBE2K active site (Fig. 1e). To better visualize the actual active site, we modeled the wild-type residues and refined the model including energy minimization. This placed the ε-amino group of K48 of Ub^A^ within ~6.4 Å of the UBE2K active site C92, suggesting that the structure approximates the transition-state complex of K48-linked Ub chain synthesis (Fig. 1f). K48 of Ub^A^ is also in close proximity (~3.6 Å) to D124 of UBE2K, suggesting a role for this residue in coordinating acceptor lysine. The proximal Ub of K48-Ub_2_ approaches the E64-T65 region of UBE2K, but does not make any contact; hence we focus the discussion on Ub^A^.

### Donor Ub is primed by RNF38 through a conserved network

**Fig. 2:**
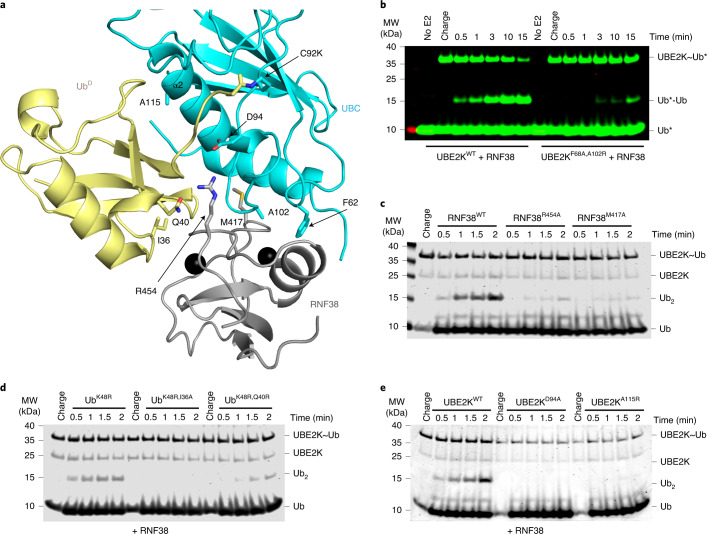
Validation of the UBE2K/Ub^D^/RING network. **a**, Structure of Ub^D^ (yellow) with the C terminus covalently attached to the UBE2K (cyan) active site K92 and primed for catalysis by RNF38 RING (gray). The isopeptide bond between UBE2K K92 and Ub^D^ G76 along with key residues in the binding interface are shown as sticks. **b**, Nonreduced SDS–PAGE showing di-Ub formation with the RING-binding-deficient UBE2K mutant. Asterisks (*) indicate fluorescent-labeled Ub. **c**, Nonreduced SDS–PAGE showing di-Ub formation in the presence of RNF38 E2/Ub^D^-binding-deficient mutants. **d**, Nonreduced SDS–PAGE showing the effect of Ub^D^ mutations on di-Ub formation with RNF38. **e**, Nonreduced SDS–PAGE of RNF38 with UBE2K^D94A^ and UBE2K^A115R^ in di-Ub formation. Coomassie staining was used to visualize **c**, **d** and **e**. Source data

**Extended Data Fig. 3:**
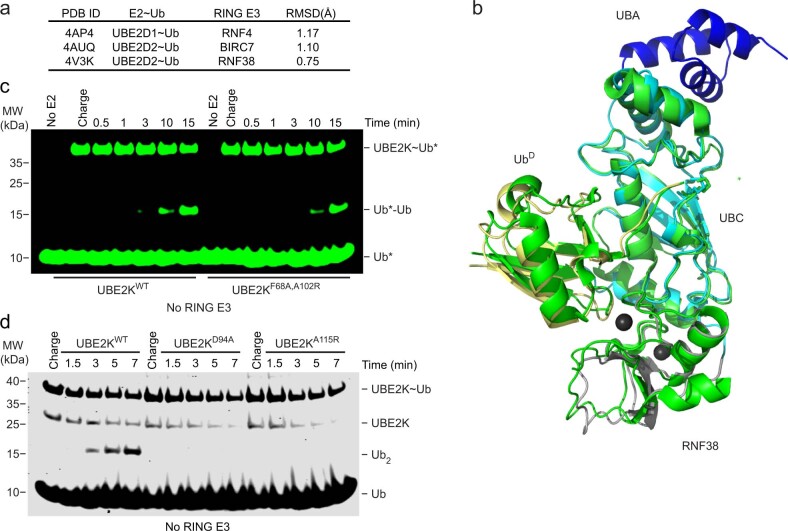
Ub^D^ in UBE2K–Ub adopts the conserved primed conformation for catalysis. (**a**) Comparison of RMSD between the RNF38-UBE2K–Ub^D^ portion of our structure and published complexes of RING E3 and E2–Ub conjugates. (**b**) Structure overlay of RNF38-UBE2K–Ub^D^ portion of structure (colored as in Fig. 1c**)** and published RNF38-UBE2D2–Ub structure (4V3K; all domains in green). (**c**) Non-reduced SDS-PAGE showing UBE2K RING binding deficient mutant in di-Ub formation assay in the absence of RNF38. UBE2K^F68A,A102R^ had a minimal effect on di-Ub formation suggesting that the E3-binding surface is dispensable for UBE2K-catalyzed ubiquitination in the absence of E3. Asterisks indicate fluorescent-labeled Ub. (**d**) Non-reduced SDS-PAGE detected with coomassie staining showing UBE2K^D94A^ and UBE2K^A115R^ mutants failed to produce di-Ub in the absence of RNF38 suggesting that the observed closed UBE2K–Ub^D^ conformation is important for E3-independent catalysis. Source data

In our complex Ub^D^ is primed in a catalytically competent conformation by RNF38 with root mean square deviations (r.m.s.d.) across Cα atoms between 0.75 and 1.17 Å compared to UBE2D2–Ub/RNF38 and other UBE2D–Ub/RING E3 complexes^25,27,28^ (Fig. 2a and Extended Data Fig. 3a,b). Furthermore, the position of the critical ‘linchpin-Arg’ R454 in RNF38 is superimposable between our structure and the UBE2D2−Ub/RNF38 complex^28^, suggesting this mechanism is conserved across E2s. To validate the importance of E2/Ub^D^/RING contacts, we made a series of mutations to UBE2K, Ub^D^ and RNF38 and assessed their performance in a di-Ub formation assay. We designed UBE2K^F62A,A102R^ and RNF38^M417A^ to disrupt the RNF38/UBE2K interaction and found both mutants impaired di-Ub formation in a RING-dependent manner (Fig. 2b,c and Extended Data Fig. 3c). The importance of RNF38’s linchpin arginine was validated using the R454A mutant, which impaired activity (Fig. 2c). Ub^D^ mutants I36A and Q40R, which were designed to disrupt the Ub^D^/RNF38 interface, were both defective compared to Ub^WT^ in our assays (Fig. 2d). UBE2K α2-helix residues are involved in forming an interface with Ub^D^, and mutation of A115 to Arg within the α2-helix prevented di-Ub formation (Fig. 2e). D94 of UBE2K contributes to priming of the C-terminal tail of Ub^D^ and D94A substitution eliminated di-Ub formation (Fig. 2e). UBE2K^D94A^ and UBE2K^A115R^ also exhibited a defect in di-Ub formation in the absence of E3 (Extended Data Fig. 3d), highlighting the importance of the primed UBE2K–Ub^D^ conformation in both E3-dependent and E3-independent reactions.

### UBC and UBA domains cooperate to stabilize acceptor Ub

**Fig. 3:**
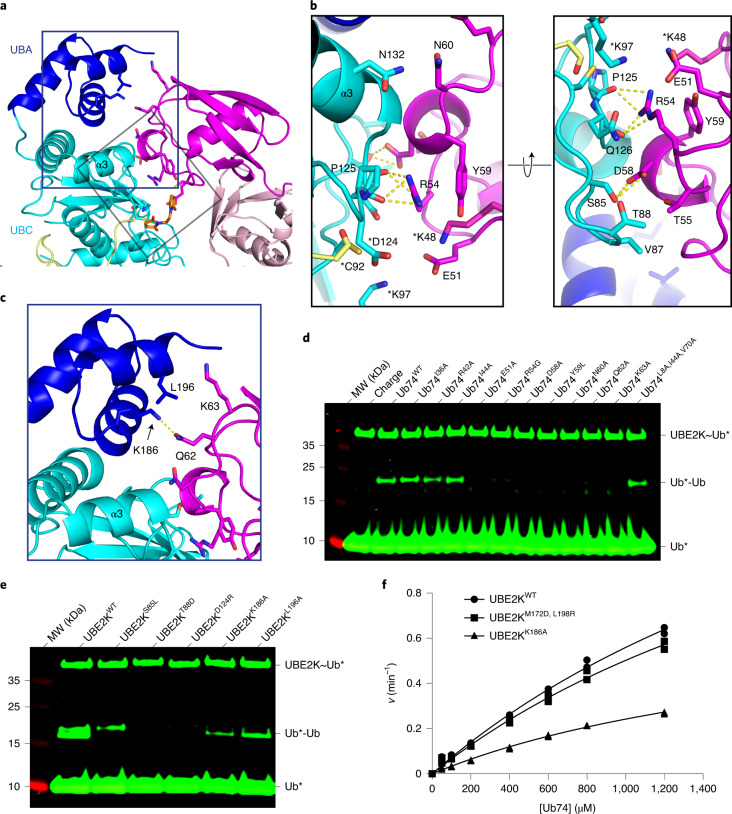
UBE2K acceptor site employs UBC and UBA to orient Ub^A^. **a**, Ub^A^ (magenta) is encased by UBE2K UBC (cyan) and UBA (blue). **b**, Close-up views of key residues (sticks) between Ub^A^ and UBC domain in **a**. Hydrogen bonds are shown as dotted lines. Asterisks (*) indicate modeled residues. **c**, Interaction between Ub^A^ and UBA domain with key residues shown as sticks. **d**, Nonreduced SDS–PAGE showing UBE2K-catalyzed di-Ub formation with Ub^A^ variants. I36, R42 and I44 are remote from the UBE2K/Ub^A^ binding interface. **e**, Nonreduced SDS–PAGE showing di-Ub formation using UBE2K with acceptor-site mutations. Asterisks (*) in **d** and **e** indicate fluorescent-labeled Ub^D^. **f**, Kinetics of di-Ub formation catalyzed by UBE2K variants. Data from two independent experiments (*n* = 2) were fitted with the Michaelis–Menten equation. *k*_cat_/*K*_m_ values (UBE2K^WT^ = 750 M^−1^ min^−1^, UBE2K^M172D,L198R^ = 684 M^−1^ min^−1^ and UBE2K^L186A^ = 355 M^−1^ min^−1^) were estimated from the slope of the linear portion of curve. Source data

**Extended Data Fig. 4:**
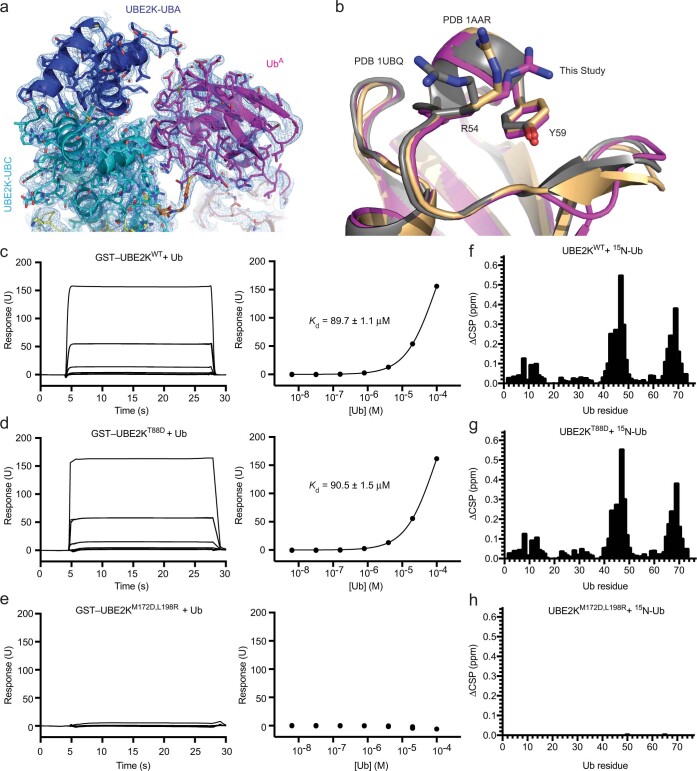
Analysis of the UBE2K/Ub^A^ binding interface. (**a**) 2Fo-Fc map (1σ) is shown at the UBE2K-Ub^A^ binding interface. (**b**) Alignment of our Ub^A^ with known structures of Ub shows a role of Y59 in selecting a conformation of R54 at the UBE2K/Ub^A^ interface. (**c-e**) SPR analysis of UBE2K and Ub binding. Sensorgrams (left) and binding curves (right) for mono-Ub and (**c**) GST-UBE2K^WT^, (**d**) GST-UBE2K^T88D^ and (**e**) GST-UBE2K^M172D,L198R^. n = 2 for each binding curve with *K*_d_ indicated where applicable. Residue specific CSPs for 200 μM ^15^N-Ub_1_ titrated until 2x molar excess of (**f**) UBE2K^WT^ and (**g**) UBE2K^T88D^, and 5x molar excess of (**h**) UBE2K^M172D,L198R^. No measurable binding is observed for UBE2K^M172D,L198R^ in either SPR or NMR. T88 is located at the UBE2K/Ub^A^ binding interface and UBE2K^T88D^ is defective in Ub^A^ catalysis (Fig. 3e), yet UBE2K^WT^ and UBE2K^T88D^ have near identical affinity for Ub. These data suggest that the canonical M172/L198 Ub binding surface contributes to the bulk of Ub binding affinity detected and that the UBE2K acceptor site has weak affinity for Ub^A^.

Ub^A^ is positioned between the UBC and UBA domains of UBE2K (Fig. 3a and Extended Data Fig. 4a). The active site cross-over loop (residues 85–90) and the N-terminal region of α3-helix (residues 124–129) from the UBC domain form a network of contacts with the short 3_10_-helix region (residues 54–60) of Ub^A^. R54 of Ub^A^ forms hydrogen bonds with carbonyl oxygens of P125 and Q126 of UBE2K. Within Ub^A^ the guanidinium group of R54 is stabilized by π stacking with Y59, a feature not observed in other structures of Ub (Fig. 3b and Extended Data Fig. 4b). The side chain and carbonyl oxygen of D58 of Ub^A^ form hydrogen bonds with the side chain of S85 and amide of A128 of UBE2K, respectively (Fig. 3b). N60 of Ub^A^ packs against A128 and N132 of UBE2K. A small hydrophobic contact is seen between V87 and T88 of UBE2K and T55 of Ub^A^ (Fig. 3b). Additionally Q62 of Ub^A^ contacts K186 in the UBA domain, while K63 of Ub^A^ abuts L196 region in the UBA (Fig. 3c). Near the active site, E51 of Ub^A^ is approaching hydrogen bond distance with K97 of UBE2K (Fig. 3b). Mutation of these key interface residues in UBE2K (S85L, T88D, D124R, K186A or L196A) or Ub^A^ (E51A, R54G, D58A, Y59L, N60A, Q62A or K63A) reduced or abolished di-Ub formation (Fig. 3d,e). These results are consistent with prior mutagenesis studies, where docking methods were used to predict UBE2K/Ub^A^ interactions^17^. The overall position of Ub^A^ in the docked model is similar to the one observed in our structure. However, our structure reveals detailed molecular interactions and a role of the UBA domain in stabilizing Ub^A^ that was not reported previously. Mutation of Ub^A^ residues remote from the acceptor site (Ub^L8A,I44A,V70A^) did not inhibit di-Ub formation (Fig. 3d). This observation suggests that Ub’s canonical I44 patch plays a minimal role at the acceptor site of UBE2K.

### UBE2K has a weak affinity for acceptor Ub

In multiple structures and studies, the UBA domain of UBE2K has been shown to bind Ub via the M172/L198 surface^17,23,29^. The nature of this interaction has been unclear since it is distant from the active site and therefore unlikely to contribute directly to Ub^A^ recognition. This UBA surface has a strong affinity for Ub, making it difficult to assess Ub binding in the acceptor site. In an effort to measure Ub^A^-binding affinity, we generated UBE2K^M172D,L198R^ to disrupt this canonical UBA/Ub interaction. Surface plasmon resonance (SPR) and NMR analysis demonstrated that UBE2K^M172D,L198R^ had no detectable binding of Ub, whereas UBE2K^T88D^, a mutant defective in Ub^A^ recognition (Fig. 3b,e), exhibited near-identical affinity for Ub as UBE2K^WT^ (Extended Data Fig. 4c–h and Supplementary Fig. 3). Furthermore, the ^1^H-^15^N HSQC spectra of ^15^N-Ub titrated with UBE2K^WT^ and UBE2K^T88D^ are nearly identical, while there were no chemical shift perturbations (CSPs) in ^15^N-Ub upon titrating UBE2K^M172D,L198R^. These data support that Ub^A^ binding is weak and the M172/L198 surface accounts for detectable Ub binding by the UBA domain. Kinetic analysis of di-Ub formation catalyzed by UBE2K^WT^ and UBE2K^M172D,L198R^ showed similar kinetics, suggesting that the M172/L198 Ub-binding site on the UBA domain has a limited role in Ub^A^ recognition and catalysis (Fig. 3f and Supplementary Fig. 4). The Michaelis–Menten curves were not saturated at concentrations of 1.2 mM Ub^A^, supporting a weak Ub^A^-binding affinity.

### NMR approach to validating UBE2K acceptor Ub site

**Fig. 4:**
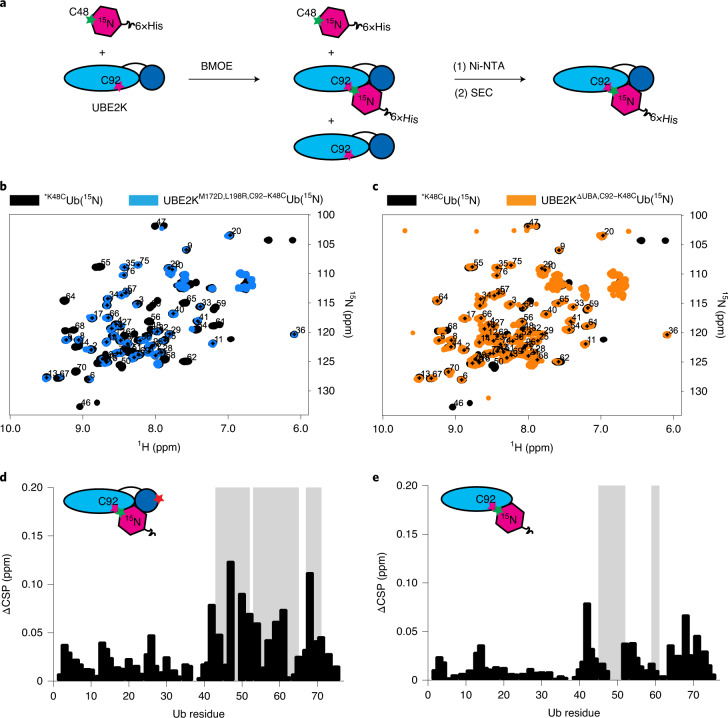
NMR analysis of Ub^A^-binding mode. **a**, Schematic of chemical cross-linking and purification scheme to obtain UBE2K^C92–K48C^Ub(^15^N) and related variants for NMR studies. **b**, Overlay of ^1^H-^15^N HSQC of UBE2K^M172D,L198R,C92–K48C^Ub(^15^N) (blue) and BMOE-reacted ^*K48C^Ub(^15^N) (black). **c**, Overlay of ^1^H-^15^N HSQC of UBE2K^ΔUBA,C92–K48C^Ub(^15^N) (orange) and BMOE-reacted ^*K48C^Ub(^15^N) (black). **d**,**e**, Residue-specific CSPs (black) and signal attenuations (gray) of ^15^N-Ub^A^ between ^1^H-^15^N HSQC spectra of UBE2K^M172D,L198R, C92–K48C^Ub(^15^N) (**d**) and UBE2K^ΔUBA, C92–K48C^Ub(^15^N) (**e**), respectively, compared with BMOE-reacted ^*K48C^Ub(^15^N). The region surrounding K63 experiences attenuations in UBE2K^M172D,L198R,C92–K48C^Ub(^15^N) but not UBE2K^ΔUBA,C92–K48C^Ub(^15^N).

**Extended Data Fig. 5:**
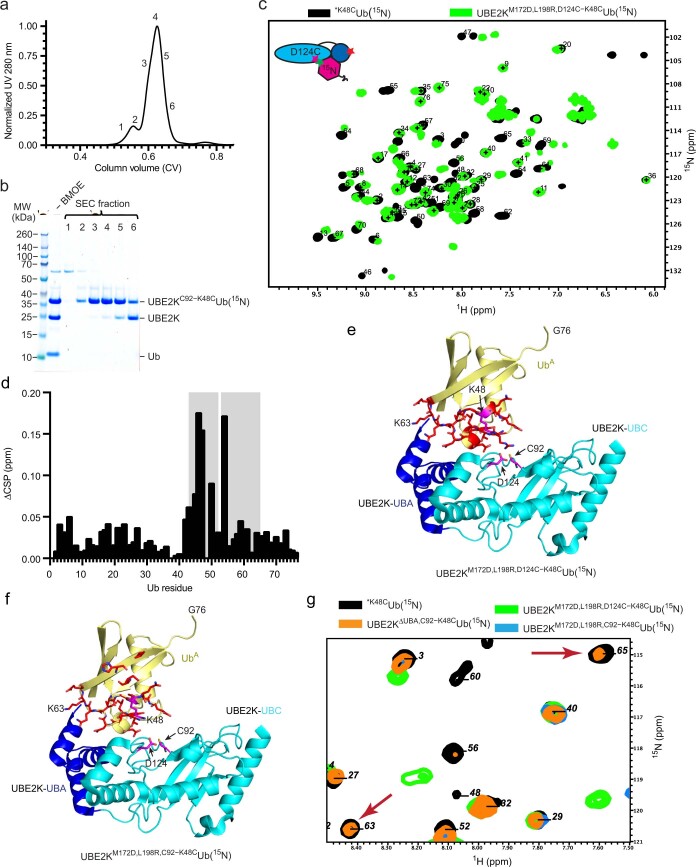
NMR analysis of UBE2K/Ub^A^ binding interface. (**a**) Superdex 75 gel filtration profile of cross-linked UBE2K^C92–K48C^Ub(^15^N) used for NMR studies and (**b**) SDS-PAGE, respectively. (**c**) Overlay of ^1^H-^15^N HSQC of UBE2K^M172D,L198R,D124C–K48C^Ub(^15^N) (green) and BMOE reacted ^*K48C^Ub(^15^N) (black). (**d**) Residue specific CSPs (black) and signal attenuations (gray bars) in UBE2K^M172D,L198R,D124C–K48C^Ub(^15^N) from **c**. (**e,f**) Our structure of Ub^A^ (yellow) at the acceptor site of UBE2K(UBC cyan and UBA blue) with signal attenuation (red sticks) mapped from **d** and (Fig. 4d), respectively. (**f**) Overlay of UBE2K^M172D,L198R,D124C–K48C^Ub(^15^N) (green), UBE2K^M172D,L198R,C92–K48C^Ub(^15^N) (blue), UBE2K^ΔUBA,C92–K48C^Ub(^15^N) (orange), and BMOE reacted ^*K48C^Ub(^15^N) (black) supports the K63 region of Ub^A^ contacts the UBA domain of UBE2K.

To validate the observed UBE2K/Ub^A^ interactions we chemically cross-linked K48 of ^15^N-Ub^K48C^ directly to the active site C92 of UBE2K to overcome the weak binding affinity of the acceptor site (Fig. 4a and Extended Data Fig. 5a,b). Cross-linking to UBE2K^WT^ still allows for canonical binding of Ub to the UBA domain, potentially leading to ambiguous signals in NMR. Therefore, M172D and L198R were incorporated such that CSPs could be unambiguously attributed to Ub^A^ binding. We generated UBE2K^M172D,L198R,C92–K48C^Ub(^15^N) and UBE2K^ΔUBA,C92–K48C^Ub(^15^N) and acquired ^15^N-^1^H HSQC spectra (Fig. 4b,c). As expected CSPs and/or signal attenuation of the signals originating from residues around K48 and residues that contact the UBC in our structure were observed for both conjugates (Fig. 4d,e). To support the suitability of our cross-linking strategy via UBE2K^D124C^ and Ub^K48C^ in our structure, we also generated UBE2K^M172D,L198R,D124C–K48C^Ub(^15^N) and found a similar ^15^N-^1^H HSQC spectrum and pattern of CSPs and signal attenuations (Extended Data Fig. 5c–f). Comparison of the spectra showed that the signal for K63 of Ub^A^ was attenuated in the presence of UBA, but not with UBE2K^ΔUBA^, corroborating our trapped mimic structure (Extended Data Fig. 5g). We attribute the reduced attenuation of the signals observed for ^15^N-Ub^A^ in UBE2K^ΔUBA,C92–K48C^Ub(^15^N) to enhanced conformational freedom of Ub^A^ in the absence of the UBA domain (Fig. 4d,e). Consistent with this notion, UBE2K^K186A^ has reduced catalytic efficiency for di-Ub formation (Fig. 3f and Supplementary Fig. 4) supporting UBA’s role in positioning Ub^A^ for catalysis. Together these results demonstrate that the UBC active site region of UBE2K functions to orient Ub^A^ to impart K48-Ub chain specificity, while the UBA domain serves to stabilize Ub^A^ to enhance catalysis.

### UBE2K is recruited to Ub-primed substrates through UBA

**Fig. 5:**
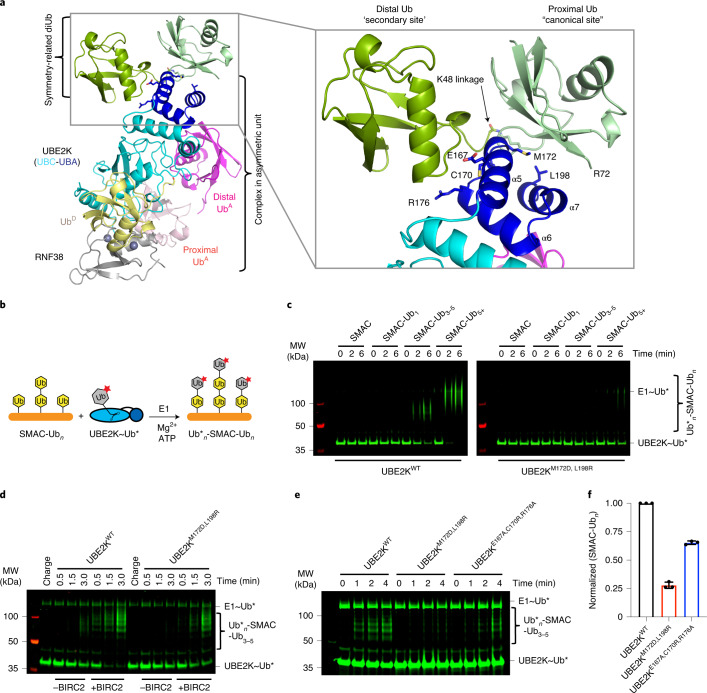
UBE2K UBA domain is critical for recruitment to Ub-primed substrates. **a**, Symmetry-related distal Ub (green) and proximal Ub (light green) of K48-Ub_2_ bind UBE2K UBA domain (blue) in *trans*. **b**, Schematic of Ub-primed substrate SMAC-Ub_*n*_ reacting with precharged UBE2K~Ub. **c** Nonreduced SDS–PAGE showing precharged UBE2K^WT^ (left) and UBE2K^M172D,L198R^ (right) transferring Ub^D^ to SMAC, SMAC-Ub_1-2_, SMAC-Ub_3-5_ and SMAC-Ub_5+_. **d**, Nonreduced SDS–PAGE showing UBE2K^WT^ and UBE2K^M172D,L198R^ catalyzed SMAC-Ub_3-5_ ubiquitination in the absence and presence of BIRC2^147-C^. **e**, Nonreduced SDS–PAGE showing UBE2K variant-catalyzed SMAC-Ub_3-5_ ubiquitination in the absence of E3. Asterisks (*) in **c, d** and **e** indicate fluorescent-labeled Ub. **f**, Plots showing normalized SMAC-Ub_*n*_ formation at the 2-min time point corresponding to **e**. Data are presented as mean value ±s.d. from three independent experiments (*n* = 3). Source data

**Extended Data Fig. 6:**
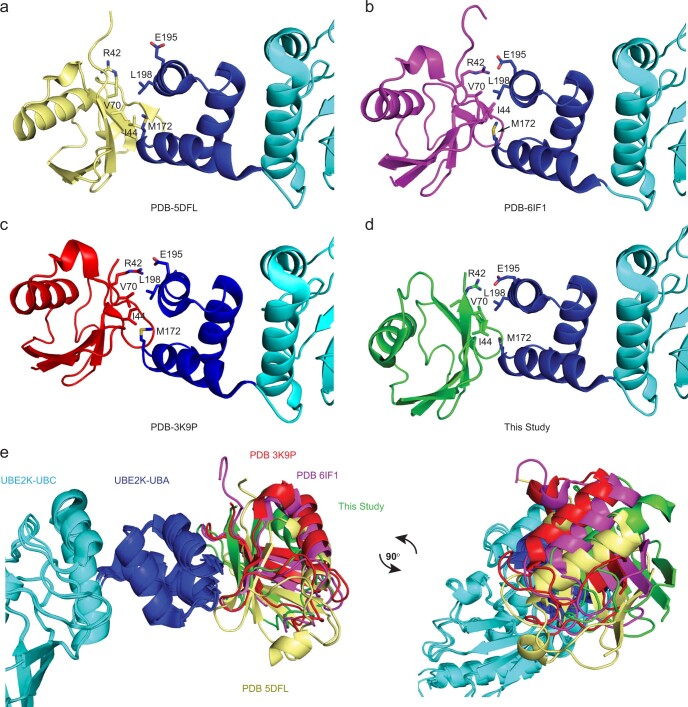
Accommodation of Ub at the M172/L198 site. (**a-d**) Structures of UBE2K/Ub complexes demonstrate a conserved interface. Hydrophobic residues M172 and L198 in UBE2K (UBC cyan and UBA blue) are in close proximity to I44 and V70 Ub, along with a polar interaction between E195 of UBE2K and R42 of Ub (each residue shown as sticks). (**e**) Structural alignment of UBE2K in complex with Ub suggests a subtle difference in binding modes.

Analysis of copies of our structure from adjacent asymmetric units (ASUs) showed a potential interaction between K48-Ub_2_ from one ASU binding in trans to the UBE2K UBA domain of another ASU, distal from the Ub^A^-binding site (Fig. 5a). Both Ub units of this neighboring K48-Ub_2_ are in close contact with the same UBA, so we investigated the role of both binding sites. The canonical Ub-binding surface centered on M172 and L198 of UBE2K contacts the I44 patch of the proximal Ub from the neighboring ASU. This interface represents a conserved binding mode between UBE2K and Ub, which can be observed in other structures with a varying degree of rotation between the two proteins (Extended Data Fig. 6). The neighboring distal Ub binds to UBE2K’s UBA α5-helix and the preceding loop (residues E167 and R176) via its I36 patch, an interaction that has not previously been reported. This resulting ‘open’ conformation of di-Ub is unique among structures of K48-linked polyUb (Fig. 5a and Extended Data Fig. 2f,g).

**Extended Data Fig. 7:**
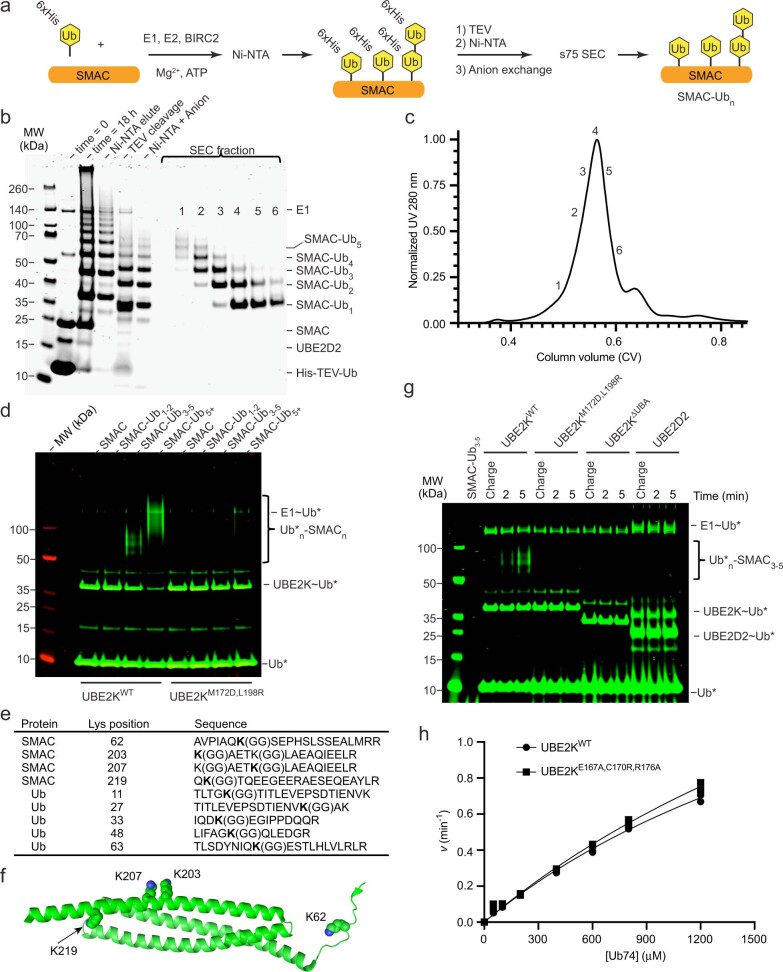
The role of UBA in facilitating UBE2K catalysis. (**a**) Schematic representing the steps involved in generation and purification of ubiquitinated SMAC substrate. His-TEV-Ub, UBE2D2, and BIRC2 are combined to obtain Ub-primed SMAC-Ub_n_. (**b**) Purification of SMAC-Ub_n_ monitored by SDS-PAGE. (**c**) Profile of the final Superdex 75 gel step confirms the isolation of SMAC modified with 1 to 5 Ubs. Fraction 2 was designated SMAC-Ub_3-5_ and served as a model Ub-primed substrate. (**d**) Non-reduced SDS-PAGE showing efficiency of Ub transfer of UBE2K^WT^ versus UBE2K^M172D,L198R^ with acceptor SMAC, SMAC-Ub_1-2_, SMAC-Ub_3-5_, or SMAC-Ub_5+_ at the 4 minute time point. Asterisks (*) indicate fluorescent-labeled Ub. (**e**) Tryptic MS analysis of SMAC-Ub_3-5_. (**f**) Ubiquitination sites K62, K203, K207, and K219 are shown as spheres on monomeric SMAC. (**g**) Non-reduced SDS-PAGE showing pre-charged E2 reacted with SMAC-Ub_3-5_ in absence of RING E3. Asterisks (*) indicates fluorescent-labeled Ub. (**h**) Comparison of Michaelis-Menten curves for di-Ub formation catalyzed by UBE2K^WT^ and UBE2K^E167A,C170R,R176A^ show similar kinetics suggesting that the mutations in the secondary Ub-binding site on UBA domain do not affect Ub^A^ binding and catalysis. Data from two independent experiments (*n* = 2) were fitted with the Michaelis-Menten equation. *k*_cat_/*K*_m_ values (UBE2K^WT^ = 829 M^-1^.min^-1^ and UBE2K^E167A,C170R,R176A^ = 847 M^-1^.min^-1^) were estimated from the slope of the linear portion of curve.

The role of UBA^M172,L198^/Ub interaction in UBE2K-catalyzed polyUb formation remains unclear. We showed that UBE2K^M172D,L198R^ had no defect in di-Ub synthesis when free unanchored Ub was used as the substrate (Fig. 3f). We wondered whether this Ub-binding surface of the UBA might serve to recruit UBE2K to ubiquitinated substrates to facilitate chain elongation. We generated a ubiquitinated substrate, SMAC-Ub_1–6_, using a combination of UBE2D2 and BIRC2 that allowed isolation of SMAC-Ub_1–2_, SMAC-Ub_3–5_ and SMAC-Ub_5+_ (Extended Data Fig. 7a–c). With this we could assess how the Ub-binding property of UBE2K’s UBA domain mediated recruitment to our model substrate, SMAC-Ub_*n*_. At a concentration of 10 μM, monomeric Ub^A^, UBE2K^WT^ and UBE2K^M172D,L198R^ exhibited minimal di-Ub formation (Fig. 3f and Supplementary Fig. 4). In contrast, at a 10-μM SMAC-Ub_*n*_ concentration, UBE2K^WT^ showed enhanced ubiquitination of SMAC modified with a minimum of three Ub molecules (Fig. 5b,c). It seems plausible that an increased number of Ub moieties on SMAC might provide more available Ub^A^ for elongation, but UBE2K^M172D,L198R^, which was as active as UBE2K^WT^ in di-Ub synthesis (Fig. 3f), showed no enhancement in ubiquitination of SMAC-Ub_3–5_ and only a marginal increase in ubiquitination with SMAC-Ub_5+_ (Fig. 5c and Extended Data Fig. 7d). These findings suggest that the UBA^M172,L198^–Ub interaction accounts for the bulk of enhanced ubiquitination in the context of Ub-primed substrates. Hereafter we selected SMAC-Ub_3–5_ for further analysis. Mass spectrometry analysis revealed that several monoubiquitination sites, as well as a variety of Ub linkage types (K11, K27, K33, K48, K63) were present, consistent with reported properties of UBE2D2 (refs. ^3,30,31^) (Extended Data Fig. 7e,f). UBE2K^WT^ catalyzed the transfer of Ub to 10 μM of SMAC-Ub_3–5_, whereas UBE2K^M172D,L198R^, UBE2K^ΔUBA^ and UBE2D2 were defective (Extended Data Fig. 7g). Given that BIRC2 stimulates UBE2K (Extended Data Fig. 1b) and binds SMAC, we performed UBE2K-mediated SMAC-Ub_3–5_ ubiquitination in the absence and presence of near-full-length BIRC2^147-C^. Addition of BIRC2^147-C^ resulted in more Ub transfer to SMAC-Ub_3–5_ by UBE2K^WT^ and partially restored the ability of UBE2K^M172D,L198R^ to transfer Ub to SMAC-Ub_3–5_ (Fig. 5d).

**Extended Data Fig. 8:**
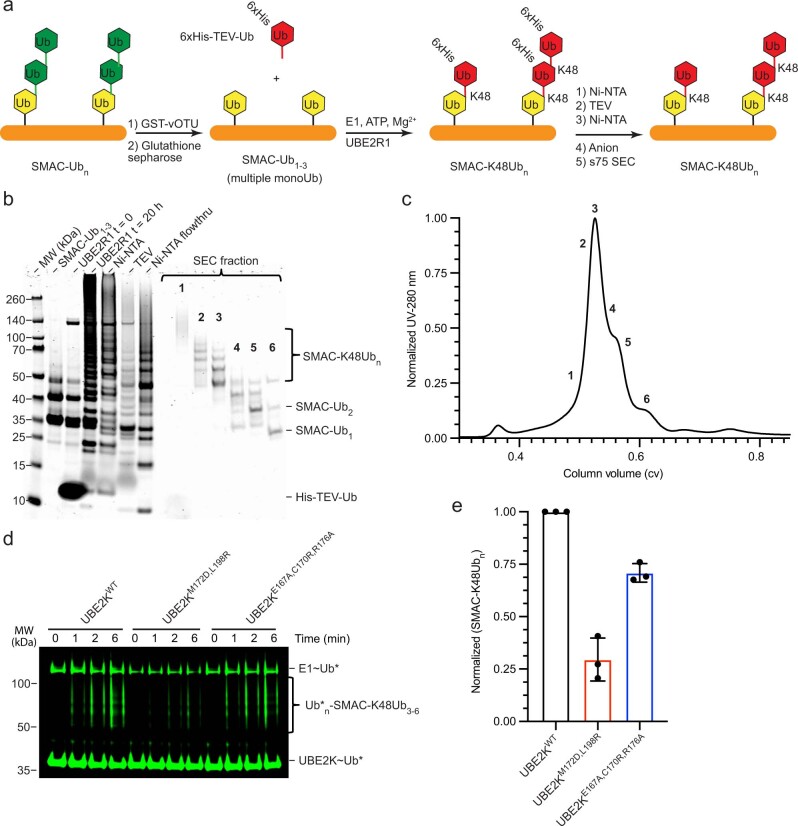
Generation of SMAC with homogeneous K48 linkages. **(a)** Synthesis and purification scheme to generate SMAC-K48Ub_n_ from multiple-mono Ub primed SMAC-Ub_1-3_. (**b**) His-TEV-Ub, UBE2R1, and BIRC2 were reacted with SMAC-Ub_1-3_ and varying lengths of SMAC-K48Ub_n_ are isolated using (**c**) final Superdex 75 gel filtration step. (**d**) Non-reduced SDS-PAGE showing ubiquitination of SMAC-K48Ub_3-6_ (Fraction 3 from **b**,**c**) catalyzed by indicated UBE2K variants. Asterisks (*) indicate fluorescent-labeled Ub. (**e**) Plots of Ub^*^ transferred to SMAC-K48Ub_3-6_ from **d**, data normalized to UBE2K^WT^ and presented as mean value ± SD from three independent experiments (n = 3). Source data

We introduced UBE2K^E167A,C170R,R176A^ to address the secondary Ub-binding site in the UBA. UBE2K^E167A,C170R,R176A^ was as competent in di-Ub synthesis as UBE2K^WT^ (Extended Data Fig. 7h and Supplementary Fig. 5), but was defective in ubiquitination of SMAC-Ub_3–5_ compared to UBE2K^WT^ (Fig. 5e,f). Comparing these UBE2K variants revealed Ub transfer to SMAC-Ub_3–5_ by UBE2K^M172D,L198R^ was reduced to ~25% of UBE2K^WT^, while UBE2K^E167A,C170R,R176A^ was reduced to ~66% (Fig. 5f). SMAC-Ub_3–5_ generated via UBE2D2 contained heterotypic Ub chain types. To eliminate unknown contributions from such Ub chains, we treated polyubiquitinated SMAC with vOTU^32^ to obtain monoubiquitinated SMAC-Ub_1–3_ and then mixed with UBE2R1 to generate SMAC-K48Ub_*n*_ and isolated SMAC-K48Ub_3–6_ for analysis (Extended Data Fig. 8a–c). Similar to SMAC-Ub_3–5_, UBE2K^M172D,L198R^ and UBE2K^E167A,C170R,R176A^ were defective in ubiquitinating SMAC-K48Ub_3–6_ compared to UBE2K^WT^ (Extended Data Fig. 8d,e). These results demonstrated that both the canonical and newly identified secondary Ub-binding sites on the UBA domain contribute to the recruitment of UBE2K to ubiquitinated substrates to facilitate Ub chain elongation.

### The UBA domain selects Ub–Ub linkage types on the acceptor

**Fig. 6:**
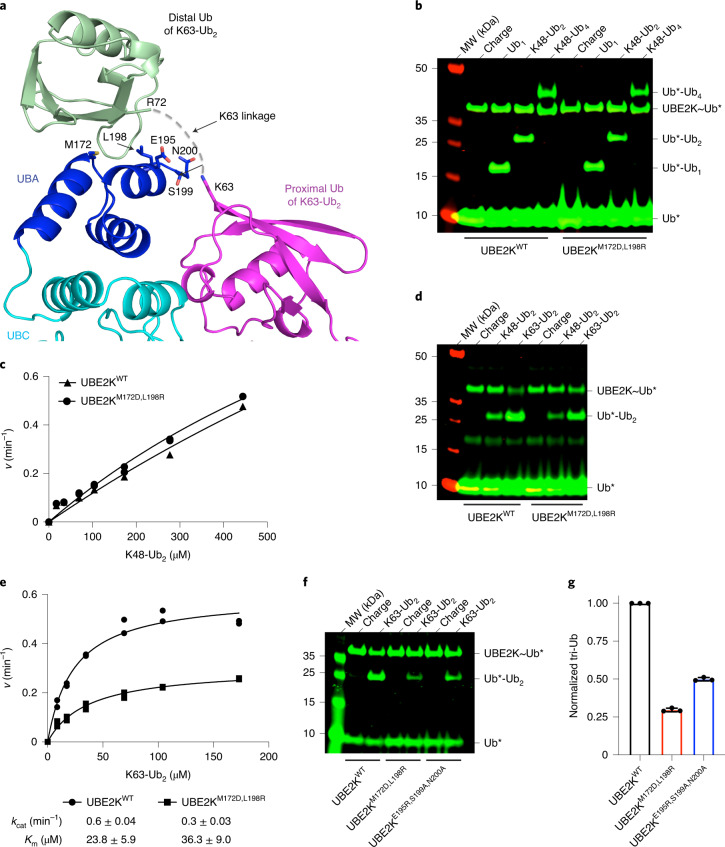
UBE2K discriminates between internal Ub linkage types. **a**, Symmetry-related Ub (light green) binds UBA (blue) with its C terminus oriented toward K63 of Ub^A^ (magenta). The potential K63 linkage is shown as a dashed line, with residues in close proximity shown as sticks. **b**, Nonreduced SDS–PAGE showing UBE2K variant-catalyzed Ub formation using different lengths of K48-linked polyUb as acceptor. **c**, Kinetics of tri-Ub formation catalyzed by UBE2K variants using K48-Ub_2_ as the acceptor. Data from two independent experiments (*n* = 2) were fitted with the Michaelis–Menten equation. *k*_cat_/*K*_m_ values (UBE2K^WT^ = 1,230 M^−1^ min^−1^ and UBE2K^M172D,L198R^ = 1,560 M^−1^ min^−1^) were estimated from the slope of the linear portion of the curve. **d**, Nonreduced SDS–PAGE showing UBE2K variant-catalyzed tri-Ub formation with K48-Ub_2_ or K63-Ub_2_ as acceptor. **e**, Kinetics of tri-Ub formation catalyzed by UBE2K variants using K63-Ub_2_ as acceptor. Data from two independent experiments (*n* = 2) were fitted with the Michaelis–Menten equation. *k*_cat_ and *K*_m_ are indicated. **f**, Nonreduced SDS–PAGE showing UBE2K variant-catalyzed tri-Ub formation using K63-Ub_2_ as the acceptor. **g**, Plots showing normalized tri-Ub product formed in **f**. Data are presented as mean value ±s.d. from three independent experiments (*n* = 3). Asterisks (*) in **b**, **d** and **f** indicate fluorescent-labeled Ub. Source data

**Extended Data Fig. 9:**
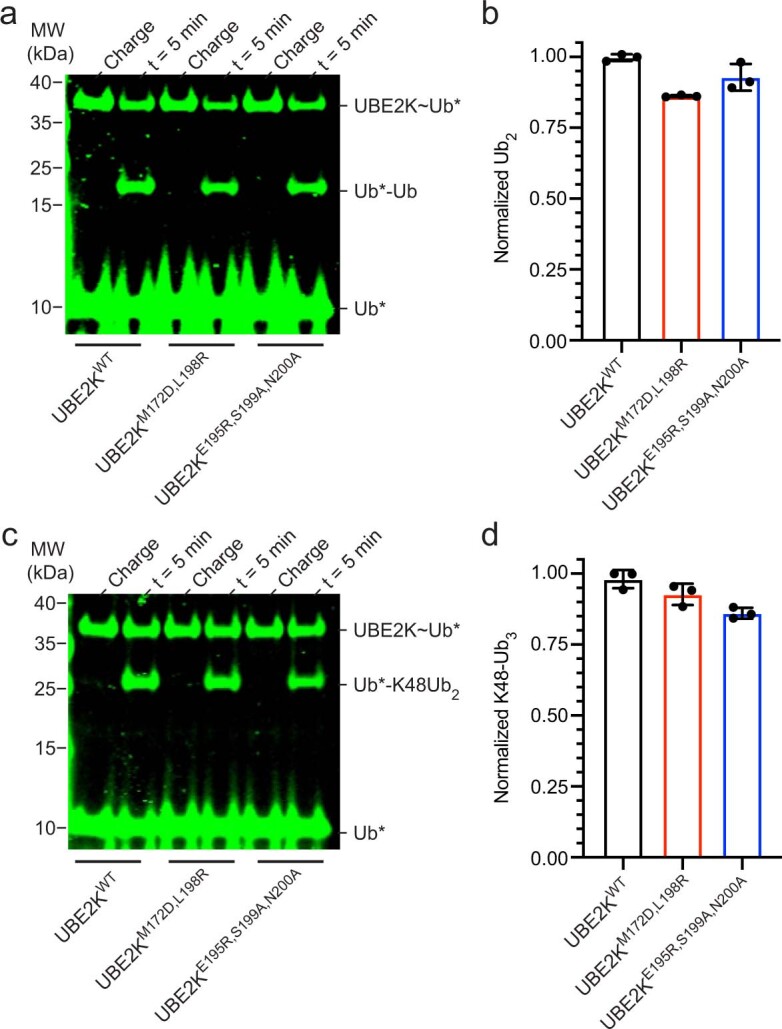
Effect of UBE2K C-terminal tail mutant. **(a)** Non-reduced SDS-PAGE showing UBE2K variant-catalyzed di-Ub formation using Ub74 as the acceptor. (**b**) Plots showing normalized di-Ub product formed in **a**. **(c)** Non-reduced SDS-PAGE showing UBE2K variant-catalyzed tri-Ub formation using K48-Ub_2_ as the acceptor. (**d**) Plots showing normalized tri-Ub product formed in **c**. Asterisks (*) in **a** and **c** indicate fluorescent-labeled Ub. In **b** and **d**, data are presented as mean value ± SD from three independent experiments (n = 3). Source data

Closer inspection of the M172/L198 Ub-binding site reveals that a plausible isopeptide linkage can be modeled between K63 of the acceptor Ub and the C terminus of a symmetry-related Ub from the M172/L198 binding site (Fig. 6a). This suggests a potential mode for binding K63-linked polyUb acceptor, which is consistent with the recently proposed model for yeast homolog Ubc1 (ref. ^33^). We examined the preference of UBE2K^WT^ and UBE2K^M172D,L198R^ for different Ub acceptors. In a single time point reaction, both UBE2K variants appeared equally competent in Ub transfer when the acceptor was mono-Ub, K48-Ub_2_ or K48-Ub_4_ (Fig. 6b). Kinetic analysis of K48-linked tri-Ub formation showed similar catalytic efficiency by both UBE2K variants (Fig. 6c and Supplementary Fig. 6a,b), suggesting that Ub/UBA binding is not a major factor for K48-linked polyUb acceptors. Similar to the situation with mono-Ub as the acceptor (Fig. 3f), the Michaelis–Menten curves could not be saturated, consistent with weak acceptor Ub-binding affinity. In contrast, both UBE2K variants showed enhanced Ub transfer with K63-Ub_2_ acceptor compared to K48-Ub_2_ acceptor (Fig. 6d). Kinetic analysis of tri-Ub formation using K63-Ub_2_ acceptor showed that UBE2K^WT^ exhibited a 20-fold higher catalytic efficiency for K63-Ub_2_ (*k*_cat_/*K*_m_ = 25,070 M^−1^ min^−1^; Fig. 6e and Supplementary Fig. 6c–e) compared to K48-Ub_2_ (estimated *k*_cat_/*K*_m_ of 1,230 M^−1^ min^−1^; Fig. 6c). This primarily results from an improved *K*_m_, since *k*_cat_ is lower for K63-Ub_2_ compared to K48-Ub_2_ at higher acceptor Ub concentration. UBE2K^M172D,L198R^ displayed reduced *k*_cat_ and *K*_m_ for K63-Ub_2_ compared to UBE2K^WT^ (Fig. 6e). The proximity of the extreme C-terminal residues E195, S199 and N200 of UBE2K to the potential K63 linkage led us to generate UBE2K^E195R,S199A,N200A^ to examine its effect (Fig. 6a). UBE2K^E195R,S199A,N200A^ exhibited similar activity as UBE2K^WT^ with mono-Ub or K48-Ub_2_ as acceptor, but was impaired with K63-Ub_2_ (Fig. 6f,g and Extended Data Fig. 9). Thus our structure provides a probable mechanism describing how K63-linked polyUb acceptor is recognized by UBE2K. Together this suggests that the canonical Ub-binding surface of UBA and the UBE2K active site could cooperate to bind two consecutive Ub moieties in K63-linked polyUb to enhance catalysis. This mechanism seems to be unique for K63-linked polyUb, as the conformations of other di-Ub linkages preclude this binding mode.

## Discussion

Our chemical biology approach allowed us to capture a transient state of UBE2K in complex with RING E3, Ub^D^ and acceptor K48-Ub_2_. Our structure and functional assays reveal multiple roles for the UBA domain, provide an explanation for how RING E3s stimulate UBE2K and demonstrate how acceptor Ub is recognized with its K48 poised for catalysis. We showed that the UBC active site region defines the selectivity for K48-linked Ub chain synthesis, while the UBA domain contributes to processive polyUb formation by orienting Ub^A^ at the active site and plays a role in recruitment to Ub-primed substrates. This is consistent with UBE2K being an elongating E2, suited to accommodate Ub rather than unmodified proteins, and reinforces the notion that diversity in regions outside the UBC domain of E2s are employed to control the Ub linkages they form.

Ligation of Ub is a transient event that necessitates chemical cross-linking to capture the structure of the transition-state complex. Previous structural studies have used three-way chemical cross-linkers to connect the active site of E2 to Ub^D^ and a lysine of the substrate, or a reactive cysteine on the E3 (refs. ^34,35^). Recently, the UBE2I–SUMO mimetic was engineered using a residue adjacent to the active site cysteine such that the catalytic cysteine of UBE2I is available for cross-linking with a lysine site (substituted as cysteine) on PCNA^36^. A similar strategy was adopted to generate a yeast CDC34^A141K^–Ub mimetic to allow chemical cross-linking of the active site cysteines of CDC34 and Uba1 (ref. ^37^). We initially adopted a similar approach but found that the UBE2K–Ub mimetic could not assume the active closed conformation. Thus we preserved the established E2–Ub amide linkage mimetic and used an active site Asp, known to engage with substrate lysine^24,25^, for cross-linking with Ub^A^. This method should be applicable for trapping other E2–Ub/E3/Ub^A^ complexes for structural analysis.

There is paucity in understanding how Ub^A^ is recognized by E2 and/or E3 to enable linkage-specific polyUb chain assembly. K63-linked and M1-linked Ub chains syntheses have been previously captured in the structures of UBE2N~Ub/UEV1 and HOIP/Ub complexes, respectively^38–41^. Both complexes showed that Ub-binding domains, such as UEV1 and a zinc finger region embedded within the catalytic domain of HOIP, are responsible for orienting Ub^A^ to confer linkage specificity. In contrast, UBE2K primarily employs residues surrounding the active site within the UBC domain to orient Ub^A^, while the UBA domain provides extra support in stabilizing Ub^A^ to enhance catalysis. This is consistent with prior observations that UBE2K^ΔUBA^ is sufficient to synthesize K48-linked Ub chains, but has a decreased rate of polyUb formation^29,42,43^. The 3_10_-helix region of Ub^A^ forms a complementary interface with the UBE2K active site. This Ub surface was previously shown to bind Rabex-5 A20 zinc finger^44^. While structural modeling of other E2s, such as UBE2R1 and UBE2D family E2s known to assemble K48-linked Ub chains, onto our structure reveals a similar complementary interface, future studies will be required to address whether other E2s utilize a similar Ub^A^-binding mode. Although our structure includes K48-Ub_2_ as the acceptor, we did not observed any interaction involving the proximal Ub. Our kinetic analysis of UBE2K^WT^-catalyzed di-Ub and tri-Ub formation using mono-Ub and K48-Ub_2_ as the acceptor, respectively, showed that tri-Ub formation was marginally faster (Figs. 3f and 6c; ~1.5-fold). Since UBE2K^WT^ and UBE2K^M172D,L198R^ exhibited similar kinetics in these reactions, this marginal increase in rate is unlikely to be due to this UBA/Ub interaction. The relevance of proximal Ub molecules in polyUb chain elongation is a poorly understood area and warrants future investigation.

In addition to the canonical Ub-binding surface, the UBA domain harbors two weak Ub-binding surfaces, which we refer to as secondary and acceptor Ub-binding sites. The role of the canonical UBA/Ub interaction in UBE2K-catalyzed polyUb chain elongation remains elusive. We showed that UBE2K has weak affinity for Ub^A^ and this probably helps to ensure that UBE2K does not assemble free unanchored Ub chains at cellular Ub concentrations. UBE2K^WT^ and UBE2K^M172D,L198R^ exhibited similar kinetics and activity in K48-linked di-Ub, tri-Ub and penta-Ub formation, suggesting that the canonical UBA/Ub interaction plays little role in unanchored K48-polyUb chain elongation. However, when substrate was primed with three or more Ub molecules on different sites that contain either K48-linked or heterogeneous Ub chains, both the canonical and secondary Ub-binding sites on the UBA domain promote Ub chain elongation on substrate. Our data suggest a model whereby these Ub-binding surfaces of the UBA domain provide avidity for Ub-primed substrates. By localizing UBE2K to Ub-primed substrates, where Ub concentration is enriched, the weak acceptor Ub affinity could be overcome. The presence of an E3 would further reduce the Ub concentration barrier by bridging UBE2K and Ub-primed substrate. We envisage that the spatial arrangement of Ub on the Ub-primed substrate probably matters, where different chain types and lengths emanating from different ubiquitination sites could influence how Ub binds the canonical, secondary and acceptor Ub-binding sites on UBA. If the arrangement permits multisite binding, the UBE2K active site could engage Ub^A^ to enhance catalysis. The presence of K63-linked Ub chain on substrate could greatly enhance catalysis by improving the *K*_m_, in agreement with a recent finding for the yeast homolog Ubc1 (ref. ^33^). Furthermore, our structural data showed that the secondary Ub-binding site could potentially support a K48-linked Ub chain. By recognizing a diverse range of Ub conjugates and orienting Ub^A^, the UBA domain is an essential feature that enables K48-linked chain extension by UBE2K. As we demonstrate in the UBE2K system, extensions beyond the UBC domain can have broad functions ranging from substrate recruitment, Ub transfer and processivity in chain extension.

## Methods

### DNA construct design

UBE2K and related variants were expressed as 6×His-SUMO fusions in pRSF-DUET1. All Ub mutants and Ub lacking C-terminal diglycines (Ub74) used in assays were expressed with an N-terminal 6×His-TEV site in pRSF-DUET1. Untagged Ub^WT^, Ub^K48C^, Ub C-terminal 6×His (Ub(6×His)) and Ub^K48C^(6×His) were expressed in pET3a and purified as described^26,45^. Human E1 (UBA1) in pET21d was a kind gift from Professor Wolberger (Addgene, plasmid 34965) and subcloned into pRSF-DUET1 with a C-terminal GGS-6×His tag. Full-length RNF138 and RING domain only MDM2^419-C^ were expressed in His-Zbasic-CL7-SUMO vectors for use in the CL7/Im7 purification system^46^. Phospho-Y371 c-CBL was obtained from an established protocol^47^. UBE4B was obtained as a His-SUMO fusion following ref. ^48^. The GST-TEV-RNF2(1–114) and His-TEV-BMI1(1–109) constructs were coexpressed to obtain the heterodimer. Full-length SIAH1 and CHIP were expressed as a His-MBP fusion and His-TEV fusion, respectively. Other RING E3s used, BIRC2^147-C^, BIRC2^556-C^, BIRC4^434-C^, RNF114, RNF8^345-C^, RNF38^389-C^ and DTX3L^544-C^ and were expressed as GST-TEV fusions in pGEX4T-1. For removal of C-terminal 6×His on Ub, YUH1 was expressed according to ref. ^26^. His-ULP1 was expressed in pET28b. SMAC^56-C,V81D^ was expressed as a SUMO fusion in pRSF-DUET1.

### Protein expression and purification

All proteins were expressed in BL-21 Rosetta 2 (DE3) chemically competent *Escherichia coli* (Novagen). LB cultures were supplemented with 1 mM MgSO_4_ and grown at 37 °C until optical density (OD_600_) of 0.8, at which the temperature was reduced to 16 °C and cultures were induced with 0.3 mM IPTG for ~16 hours. Cultures of RING E3s were supplemented with 400 μM ZnSO_4_ 30 min before induction. Cells were collected at 3,500*g* and resuspended in IMAC buffer A (20 mM Tris–HCl, 500 mM NaCl, 1 mM TCEP, pH 7.4), lysed at 12,000 psi using a microfluidizer, cleared at 18,000*g* for 45 min and the supernatant was passed through a 0.45-μm syringe filter before loading on an affinity column. His-SUMO, His-MBP or His-TEV proteins were loaded on a 5-ml His-Trap column (GE Life Sciences), washed for 30 column volume (CV) in IMAC buffer A and eluted in IMAC buffer B (20 mM Tris–HCl, 500 mM NaCl, 300 mM imidazole, 1 mM TCEP, pH 7.4). GST-tagged proteins were loaded on a 5-ml GST trap column (GE Life Sciences), washed for 20 CV and eluted in GST elution buffer (20 mM Tris–HCl, 500 mM NaCl, 1 mM TCEP, 20 mM reduced glutathione, pH 8.0). Elutions were dialyzed against 20 mM Tris–HCl, 200 mM NaCl, 0.5 mM TCEP, pH 8.0 overnight at 4 °C. SUMO constructs were cleaved using 5 μM ULP1 and TEV cleavage carried out at 1:50 (mg/mg) ratio of TEV protease/protein. Protease was removed using a 15-ml His-Trap column before a final size-exclusion step on a HiLoad 26/600 Superdex 75 column (GE Life Sciences) in SEC buffer (20 mM Tris–HCl, 200 mM NaCl, 0.5 mM TCEP, pH 7.5). CL7 fusions were loaded on an Im7 column (TriAltus Bioscience), washed in 30 CV in CL7 buffer (20 mM Tris–HCl, 700 mM NaCl, 1 mM TCEP, pH 7.5) and cleaved on-column by injecting 1 mg of ULP1 per ml of Im7 resin allowing for a one-hour incubation. Proteins were directly loaded onto a HiLoad 26/600 Superdex 75 column from the Im7 column in SEC buffer and ULP1 was removed by addition of Ni-NTA. Human E1 was subjected to anion exchange and a final size-exclusion step on a HiLoad 26/600 Superdex 200 column (GE Life Sciences) in SEC buffer. ^15^N-Ub^K48C^(6×His) was expressed with ^15^N-ammonium chloride (Cambridge Isotope Laboratories) as the sole nitrogen source in autoinducing media according to ref. ^45^.

### PolyUb conjugation and purification

K48-linked polyUb conjugates were obtained from reaction with UBE2R1. Ub^K48C^ (0.8 mM) and 1 mM of Ub(6×His) were reacted with 20 μM UBE2R1, 3 mM TCEP, 20 mM ATP, 20 mM MgCl_2_ and 1 μM UBA1 in 50 mM Tris–HCl, pH 8.0 at 30 °C for 18 hours. ^K48C^Ub−^48^Ub(6×His) was purified using a 5-mL His-Trap followed by a HiLoad 26/600 Superdex 75 column in 25 mM Tris–HCl, 300 mM NaCl and 3 mM TCEP, pH 7.5. Unanchored wild-type K48-linked conjugates were formed under the same reaction conditions using 1 mM Ub^WT^ and 0.8 mM His-TEV-Ub^ΔGG^, followed by Ni-NTA pulldown, TEV cleavage and an additional Ni-NTA step to remove TEV protease. The final cation exchange step used a 0–35% gradient buffer A (50 mM ammonium acetate, pH 4.5) and buffer B (50 mM ammonium acetate, 500 mM NaCl, pH 4.5) over 60 CV on a 5-mL HiTrap SP HP (GE Life Sciences) to separate Ub_1_ to Ub_4_. Unanchored wild-type K63-linked polyUb was obtained from identical conditions substituting UBE2R1 for 20 μM UBE2N/UBE2V2.

### UBE2K–Ub chemical cross-linking

A 50 μM portion of UBE2K^C92K,K97R,D124C,C170S^ was loaded with 1 mM His-TEV-Ub^WT^ and 3 μM E1 in 50 mM Tris–HCl, 20 mM ATP, 20 mM MgCl_2_, 1 mM TCEP, pH 9.5 buffer at 30 °C for 20 hours. The His-TEV-Ub–^C92K^UBE2K^D124C^ product was isolated in a Ni-NTA step, cleaved with TEV and the protease was removed using another Ni-NTA step. The Ub–^C92K^UBE2K^D124C^ intermediate was further purified using a HiLoad 26/600 Superdex 75 column in 20 mM Tris–HCl, 200 mM NaCl, 5 mM TCEP, pH 7.5. In the next phase, 20 mM TCEP was added to ^K48C^Ub−^48^Ub(6×His) before loading on a HiPrep 26/10 Desalting column (GE Life Sciences) in cross-linking buffer (25 mM sodium phosphate, 200 mM NaCl, 2 mM EDTA, pH 7.0). Fractions containing the fully reduced ^K48C^Ub−^48^Ub(6×His) were collected and 15-fold molar excess of BMOE was immediately added. Ub–^C92K^UBE2K^D124C^ was reduced in 20 mM TCEP and desalted, while BMOE was reacted ^K48C^Ub−^48^Ub(6×His) desalted directly into reduced Ub–^C92K^UBE2K^D124C^ and allowed to react at ambient temperature for 4 hours. This cross-linking reaction was diluted 10-fold in 25 mM Tris–HCl, 500 mM NaCl, 3 mM β-mercaptoethanol (BME), pH 7.5 buffer and loaded onto a 5-mL His-Trap column. After loading, the column was washed for 30 CV in IMAC buffer A, and eluted in 5 CV using IMAC buffer B. The elution was concentrated in a 10,000 MWCO centrifugal filter unit (Merck-Millipore) and loaded onto a HiLoad 26/600 Superdex 75 column in 20 mM Tris–HCl, 200 mM NaCl, pH 7.5. For NMR UBE2K^C92−K48C^Ub(^15^N) and UBE2K^D124C−K48C^Ub(^15^N) conjugates were created following the same procedure, but 25 mM sodium phosphate, 120 mM NaCl, pH 7.0 buffer was used in the final gel filtration step. Protein concentration was determined by Bio-Rad protein assay or by using molar extinction coefficient at 280 nm (*ε*_280_) of 22,500 cm^−1^ M^−1^ for UBE2K and *ε*_280_ = 1,490 cm^−1^ M^−1^ for Ub.

### Crystallization

For crystallization of the complex, Ub–^C92K^UBE2K^D124C−K48C^Ub−^48^Ub and RNF38 RING protein solutions were mixed at a 1:1.2 molar ratio, at a total complex concentration of about 5 mg/ml^−1^. The crystals were grown in Molecular Dimensions screen PACT-premier HT-96, condition G11 (0.2 M sodium citrate, 0.1 M Bis–Tris propane 7.5 and 20% (w/v) PEG 3350). The crystallization drops were set as a 1:1 mixture of the protein complex solution and the precipitant solution. The crystals were grown at 19 °C using vapor diffusion and sitting-drop set-up. Crystals were subsequently subjected to dehydration performed by gradually increasing the concentration of PEG 3350 in the well solution, from the initial 20% (w/v) up to 40% (w/v), using 5% increments every 2–3 days. Before data collection the crystals were cryo-protected in LV CryoOil solution (MiTeGen), then immediately cryo-cooled in liquid nitrogen.

### X-ray data collection, structure determination and refinement

Diffraction data were collected at the Diamond Light Source beam line I04-1 (0.9119 Å, 100 K), and processed with DIALS^49^ and the CCP4 program suite^50^ at a resolution of 2.4 Å. Initial phases were obtained by molecular replacement with Phaser^51^ using structures of UBE2K (PDB 1YLA), Ub (PDB1UBQ) and RNF38 RING domain (PDB 4V3L) as search models. Refinement was performed using Coot^52^ and Phenix^53^. Final Ramachandran statistics: favored: 94.75%, allowed: 5.25%, outliers: 0%. Details of the structure determination and refinement statistics are summarized in Supplementary Table 1. All figure models were generated using PyMOL (Schrödinger). Protein sequence alignments were made with MUSCLE^54^ and displayed in Jalview^55^. Modeling of the native structure and energy minimization was done using GalaxyRefineComplex^56^.

### Synthesis and purification of SMAC-Ub_*n*_ conjugates

Defined SMAC-Ub_*n*_ conjugates were generated from a reaction of 100 μM SMAC^56-C,V81D^ with 1 mM His-TEV-Ub^WT^, 20 μM UBE2D2, 2 μM E1, 3 μM BIRC2^147-C^, 20 mM ATP, 20 mM MgCl_2_ and 3 mM TCEP in 50 mM Tris–HCl pH 8.0 at 30 °C for 18 hours. The reaction was diluted 1:100 (v/v) in IMAC buffer A, loaded on a 5-ml His-Trap column, washed in 30 CV, eluted with IMAC buffer B and dialyzed in 20 mM Tris–HCl, 200 mM NaCl, pH 7.5 with TEV protease at 4 °C for 16 hours. This was passed through a His-Trap column following TEV cleavage, the flow-through was diluted 1:20 (v/v) in 50 mM Tris–HCl pH 8.9, loaded on a HiTrap Q HP anion exchange column (GE Life Sciences), washed for 20 CV and eluted in one step with 50 mM Tris–HCl, 500 mM NaCl, pH 7.5. SMAC-Ub_1–6_ was resolved using a HiLoad 26/600 Superdex 75 column in running SEC buffer (Extended Data Fig. 7a–c). To generate SMAC-K48Ub_*n*_, SMAC-Ub_*n*_ was reacted with GST-vOTU^32^ to remove residual polymeric Ub to generate multiple monoubiquitinated SMAC-Ub_1–3_ and the DUB was removed by a glutathione-sepharose purification step. A 75 μM portion of primed monoubiquitinated SMAC-Ub_1–3_ was reacted as above, except for the omission of BIRC2 and substitution of UBE2D2 for UBE2R1. SMAC-K48Ub_*n*_ was obtained through the procedure above and defined lengths were isolated in the final size-exclusion step (Extended Data Fig. 8a–c).

### Conjugation of near-infrared fluorescence labeled Ub

Reduced GGSC-Ub^K48R^ was exchanged into cross-linking buffer using a Zeba Spin desalting column (Thermo Fisher Scientific). IRDye 800CW Maleimide (LI-COR Biosciences) was resuspended in DMSO and added at a threefold molar excess to GGSC-Ub^K48R^ and allowed to incubate at room temperature for 90 min. Labeling was quenched with the addition of 3 mM BME and excess dye was removed and buffer-exchanged to 25 mM Tris–HCl, 150 mM NaCl, pH–8.0 using a using a Zeba Spin desalting column. The final stock 200 μM of infrared-labeled GGSC-Ub^K48R^ (Ub^*^) was mixed with unlabeled Ub^K48R^ in a 1:80 molar ratio to obtain a working stock.

### Kinetic analysis of di-Ub and tri-Ub formation

Ub^K48R^ was used to charge all UBE2K variants to prevent the labeled donor Ub from acting as the acceptor. ^32^P-Ub^K48R^ was prepared as described previously^47^. The charged reactions were performed in 50 mM Tris–HCl, pH 7.6, 50 mM NaCl, 10 mM ATP, 10 mM MgCl_2_, 1 mg ml^−1^ BSA, 3 μM UBA1, 9 μM UBE2K variant and 13.5 μM ^32^P-Ub K48R at 37 °C for 30 min followed by equilibration at 22 °C for 10 min. A 6-μl portion of charged reaction was added to 12 μl of chased reaction containing the indicated final concentrations of Ub74 (Fig. 3f, Extended Data Fig. 7h and Supplementary Figs. 4 and 5), K48-linked di-Ub (Fig. 6c and Supplementary Fig. 6a,b) or K63-linked di-Ub (Fig. 6e and Supplementary Fig. 6c,d) at 22 °C and stopped at the indicated time (30 s or 1 min) by adding 6 μl of the reaction to 2× LDS loading buffer. Under these reaction conditions ~10–15% of UBE2K~^32^P-Ub was consumed at the highest acceptor Ub concentration, and less than 10% at other concentrations. Thus, the reaction satisfied the initial rate requirements for kinetic analysis. The reactions were resolved by SDS–PAGE, dried, exposed to a phosphorimager, scanned with a Typhoon PLA 7000 (GE Healthcare) and quantified using ImageQuant (GE Healthcare). Known amounts of ^32^P-Ub^K48R^ were resolved by SDS–PAGE, dried and analyzed together with the reaction products. Di-Ub or tri-Ub products and charged UBE2K~^32^P-Ub were quantified. Initial rate is expressed as moles of di-Ub or tri-Ub formed per minute per moles of UBE2K~^32^P-Ub or per minute. All reported kinetic parameters were determined from initial rates by fitting two independent datasets to the Michaelis–Menten equation using Prism (GraphPad). In cases where Michaelis–Menten curves were not saturated, *k*_cat_/*K*_m_ was estimated from the slope of the linear portion of the curve.

### Continuous ubiquitination assays

RING stimulation of UBE2K (Fig. 1a and Extended Data Fig. 1d) was carried out in assay buffer (25 mM Tris–HCl, 50 mM NaCl, pH 8.0) with 15 mM ATP, 15 mM MgCl_2_, 1 μM UBA1, 200 μM Ub^WT^ and 5 μM UBE2K at 37 °C. The concentration of E3s was kept at 5 μM. UBE2K D124 variants (Fig. 1b) were assessed under identical conditions. Reactions were stopped at indicated time points with LDS loading buffer and resolved by SDS–PAGE. Gels were transferred to nitrocellulose membrane (Bio-Rad) using the Trans-Blot Turbo system (Bio-Rad) for 10 min with constant 1.3 amps. Total protein was visualized with Ponceau-S, imaged using Bio-Rad ChemiDoc, destained and the membrane was blocked in 5% (w/v) BSA in TBST (20 mM Tris–HCl, 150 mM NaCl, 0.1%(w/v) Tween-20) for 30 min at ambient temperature. Primary antibodies were incubated at 4 °C for 12 hours using a 1:5,000 dilution of mouse anti-Ub (PD41, Santa Cruz Biotechnology) and 1:3,000 dilution of rabbit anti-K48-Ub (clone Apu2, Merck) in 2.5% (w/v) BSA in TBST. The membrane was washed three times for 5 min in TBST and incubated with secondary antibodies: IRDye 800CW goat anti-mouse IgG (LI-COR Biosciences) in a 1:15,000 dilution and IRDye 680RD goat anti-rabbit IgG (LI-COR Biosciences) in a 1:15,000 dilution, for 1 hour at ambient temperature. Two washes with TBST and a final in TBS were carried out before imaging on a LI-COR Odyssey CLx.

### Precharged ubiquitination assays

Charging of UBE2K was carried out in assay buffer with 20 mM ATP, 20 mM MgCl_2_, 3 μM UBA1, 18 μM Ub^*^ and 10 μM UBE2K for 30 min at 37 °C. UBE2K^ΔUBA^ required 60 min for optimal charging. For unlabeled Ub^D^ the same concentration was used. The charging reaction of UBE2K~Ub^*^ was diluted 1:1 into the acceptor Ub conjugate to start each reaction. The final acceptor concentration of mono-Ub was 100 μM for Figs. 2c–e and 3d,e and Extended Data Figs. 3c,d and 9a. K48-Ub_2_ as acceptor was used in a final concentration of 50 μM for Fig. 6b,d and Extended Data Fig. 9c, while K63-Ub_2_ as acceptor was used at 40 μM for Fig. 6d,f. The final SMAC-Ub_*n*_ concentration was 10 μM. The reactions were stopped with LDS loading buffer and resolved by SDS–PAGE. In-gel fluorescence was detected using an Odyssey CLx imaging system and analyzed with ImageStudio v.5 and the Fiji distribution of ImageJ^57^. Total protein was detected with InstantBlue (abcam) and Coomassie-stained gels were imaged using a LI-COR Odyssey CLx imaging system and a conventional scanner.

### pH-dependent di-Ub formation assay

Buffers covering pH 6.5 to 10.5 were prepared in 50 mM Tris–HCl and150 mM NaCl at the desired pH. Charging of 30 μM UBE2K was carried out in 20 mM Tris–HCl, 100 mM NaCl, 20 mM ATP, 20 mM MgCl_2_, pH 8.0 buffer with 3 μM UBA1 and 25 μM Strep-Tag II-Ub^K48R^ for 30 min at 37 °C. The charging reaction of UBE2K~Ub was diluted 10-fold into the indicated pH buffer with a final concentration of 100 μM Ub_74_ as acceptor to begin the reaction. Samples were taken at 2 min, mixed with LDS loading buffer and resolved by SDS–PAGE. Protein gels were transferred to nitrocellulose membranes as described in the section on ‘Continuous ubiquitination assays’ and incubated with the mouse anti-Strep-Tag II (Merck) primary antibody in a 1:2,000 dilution overnight. The secondary IRDye 800CW goat anti-mouse IgG (LI-COR Biosciences) in a 1:15,000 dilution was used to detect Strep-Tag II-Ub^K48R^ using a LI-COR Odyssey CLx. The intensity of the di-Ub band was calculated as a fraction of total Strep-Tag II-Ub^K48R^ following a similar approach^41^ and the p*K*_a_ was determined from sigmoidal standard titration curve (equation (1), Prism) where *η*_H_ is the Hill slope and minUb_2_ and maxUb_2_ correspond the minimum and maximum fraction of Ub_2_ formation, respectively.

1 $${\mathrm{fraction}}\,{\mathrm{Ub}}_2 = \min {\mathrm{Ub}}_2 + \frac{{{\mathrm{maxUb}}_2 - {\mathrm{minUb}}_2}}{{1 + 10^{({\mathrm{p}}K_{\mathrm{a}} - {\mathrm{pH}}) \eta _{\mathrm{H}}}}}$$

### Solution NMR experiments

NMR data were acquired on a Bruker Avance III 600 MHz spectrometer with a cryogenic triple resonance inverse probe. UBE2K conjugates were exchanged into NMR buffer (20 mM sodium phosphate, 100 mM NaCl, 7.5% D_2_O, pH 7.0) at a final concentration of 50 μM. ^1^H-^15^N HSQC spectra were recorded at 298 K with 128 scans and 64 complex points using a sweep width of 36 parts per million (ppm) in the ^15^N dimension. All spectra were processed with 256 points in the indirect dimension using Bruker TopSpin v.3.5 patch level 7 and analyzed using CARA and NMRFAM-SPARKY^58^.

CSPs were calculated using the following:

$${\mathrm{CSP}} = \left[ {\left( {\delta _{\mathrm{HA}} - \delta _{\mathrm{HB}}} \right)^2 + \left( {\left( {\delta _{\mathrm{NA}} - \delta _{\mathrm{NB}}} \right)/5} \right)^2} \right]^{1/2}$$

where, for a given residue at state A and B, δ_H_ and δ_N_ are the difference in proton and nitrogen chemical shifts, respectively.

### SPR binding and analysis

SPR binding experiments were performed at 25 °C on a Biacore T200 instrument using a CM-5 chip (GE Healthcare) with coupled anti-GST antibody as described previously^48^. GST-tagged UBE2K variants were coupled on the chip and a serial dilution of Ub in running buffer containing 25 mM Tris–HCl, pH 7.6, 150 mM NaCl, 1 mM DTT and 0.005% (v/v) Tween-20 was used as analyte. Two technical replicates were performed and data were analyzed with BIAevalution (GE Healthcare) and fit to a one-site binding model using Prism (GraphPad).

### Statistics and reproducibility

Figure 1a,b was independently reproduced three times with similar results and the representative western blot is presented. Similar results were produced across two independent experiments for Figs. 2b–e, 3d,e, 5c,d and 6b,d with the representative In-gel or Coomassie shown. The anti-Ub western blots in Extended Data Fig. 1b were reproduced across three independent experiments with the representative blots displayed. For Extended Data Fig. 2d, generation of the Ub–^C92K^UBE2K^D124C−K48C^Ub−^48^Ub complex was reproduced across two independent purifications. For Extended Data Fig. 3c,d the same trend of di-Ub formation was observed across three independent experiments. For Extended Data Fig. 5b, purification of cross-linked UBE2K^C92–K48C^Ub(^15^N) used for NMR studies was reproduced across three independent cross-linked variants. For Extended Data Figs. 7b and 8b, generation of SMAC-Ub_*n*_ and SMAC-K48Ub_*n*_, respectively, were reproduced across two independent purifications. The same trend for SMAC-Ub_*n*_ conjugates in Extended Data Fig. 7d was observed across three independent experiments.

### Reporting Summary

Further information on research design is available in the Nature Research Reporting Summary linked to this article.

## Online content

Any methods, additional references, Nature Research reporting summaries, source data, extended data, supplementary information, acknowledgements, peer review information; details of author contributions and competing interests; and statements of data and code availability are available at 10.1038/s41589-021-00952-x.

## Supplementary information

Supplementary Information Supplementary Figs. 1–9 and Table 1

Reporting Summary

## Data Availability

Coordinates and structure factors have been deposited with the protein data bank under accession code 7OJX. The data that support the findings of this study are available within the paper and its supplementary information file. Source data are provided with this paper. DNA constructs are available upon request from the corresponding author.

## Source data

Source Data Fig. 1 Unprocessed gels for Fig. 1.
Source Data Fig. 2 Unprocessed gels for Fig. 2.
Source Data Fig. 3 Unprocessed gels for Fig. 3.
Source Data Fig. 5 Unprocessed gels for Fig. 5.
Source Data Fig. 6 Unprocessed gels for Fig. 6.
Source Data Extended Data Fig. 1 Unprocessed gels for Extended Data Fig. 1.
Source Data Extended Data Fig. 3 Unprocessed gels for Extended Data Fig. 3.
Source Data Extended Data Fig. 8 Unprocessed gels for Extended Data Fig. 8.
Source Data Extended Data Fig. 9 Unprocessed gels for Extended Data Fig. 9.
