# Supplementary material for: Structure of UBE2K–Ub/E3/polyUb reveals mechanisms of K48-linked Ub chain extension
Source: Nat Chem Biol. 2022 Jan 13;18(4):422–31. doi: 10.1038/s41589-021-00952-x (PMC8964413; doi:10.1038/s41589-021-00952-x)
Supplement: Supplementary file 1 — Supplementary Figs. 1–9 and Table 1 [file 41589_2021_952_MOESM1_ESM.pdf]

nature portfolio

https://doi.org/10.1038/s41589-021-00952-x

Supplementary information

Structure of UBE2K–Ub/E3/polyUb reveals mechanisms of K48-linked Ub chain extension

In the format provided by the authors and unedited

Supplemental Information

Structure of UBE2K–Ub/E3/polyUb reveals mechanisms of K48-linked Ub chain extension

Mark A. Nakasone, Karolina A. Majorek, Mads Gabrielsen, Gary J. Sibbet, Brian O. Smith, and Danny T. Huang

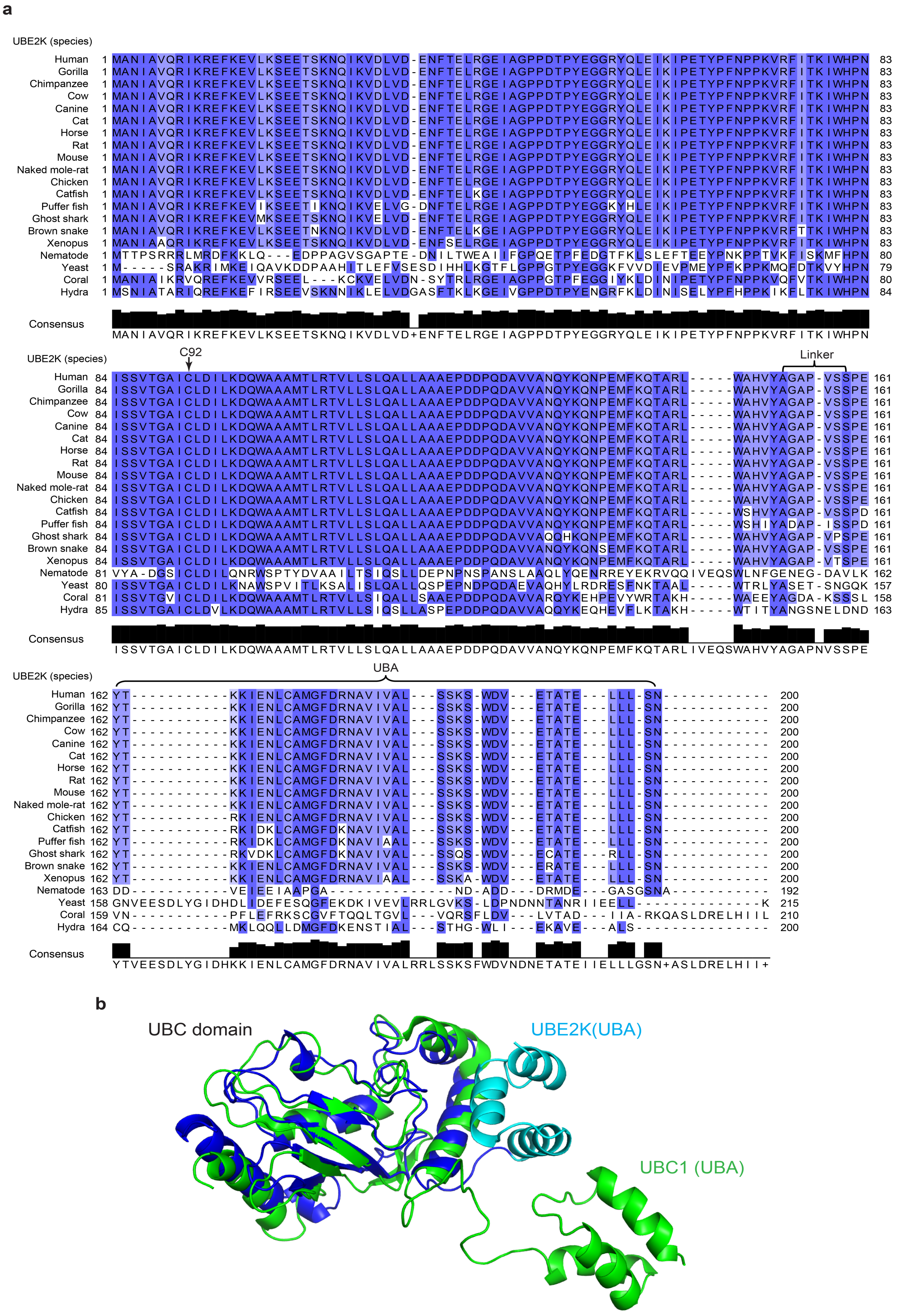

Supplementary Figure 1. **Domain structure of UBE2K**. (**a**) Sequence alignment of UBE2K across species with consensus (black bars). (**b**) Structural superposition of human UBE2K (PDB 1YLA; UBC in cyan and UBA in blue) with yeast homologue Ubc1 (PDB 1TTE; green).

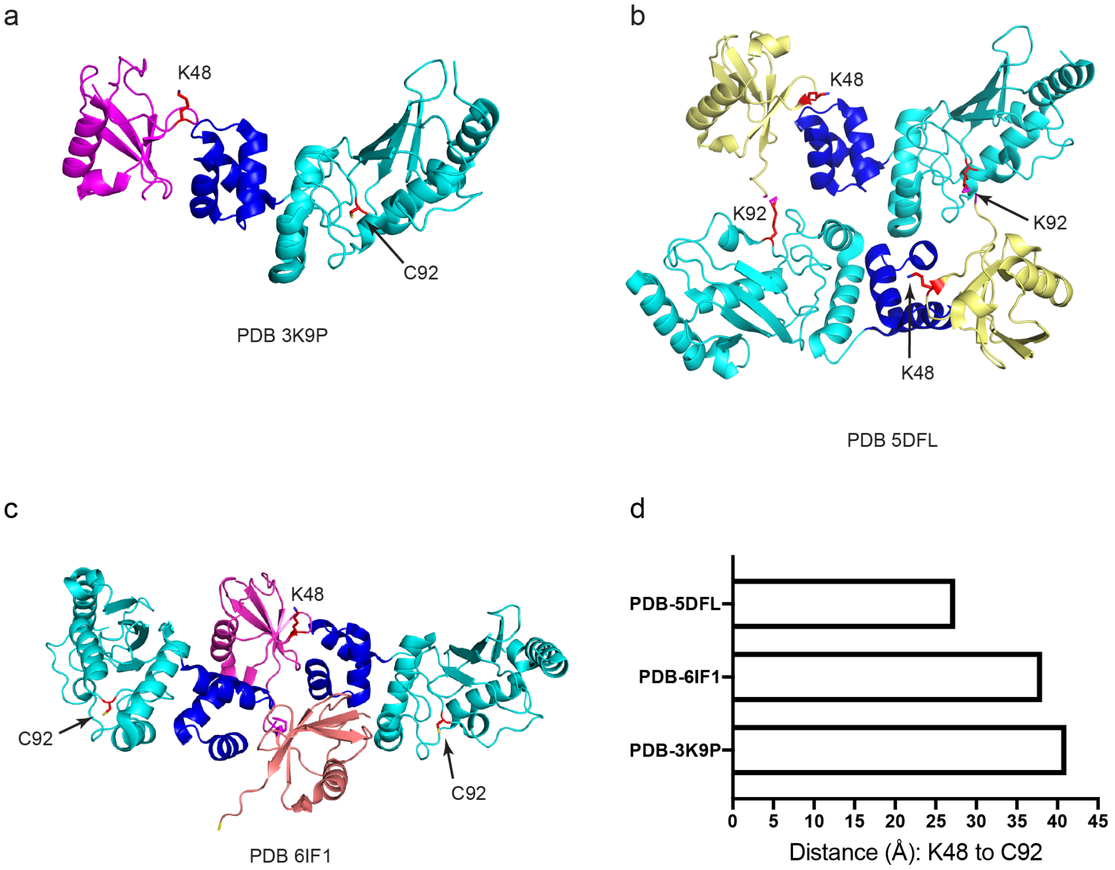

Supplementary Figure 2. **Published structures of UBE2K and Ub complexes**. (**a**) Structure of UBE2K (cyan) bound to mono-Ub (magenta) on the M172/L198 binding site (PDB-3K9P). (**b**) Structure of UBE2K^{C92K} (cyan) loaded with Ub^{D} (yellow), where Ub binds the M172/L198 site *in trans* (PDB-5DFL). (**c**) Structure of a non-covalent complex of UBE2K and K48-Ub$_2$ where the distal (magneta) and proximal (salmon) units in K48-Ub$_2$ bind separate UBE2K molecules on the M172/L198 site (PDB-6IF1). UBE2K catalytic C92 and Ub K48 are indicated. (**d**) The shortest distance between Cβ in residue 92 of UBE2K and Cβ of K48 in Ub is plotted for each structure.

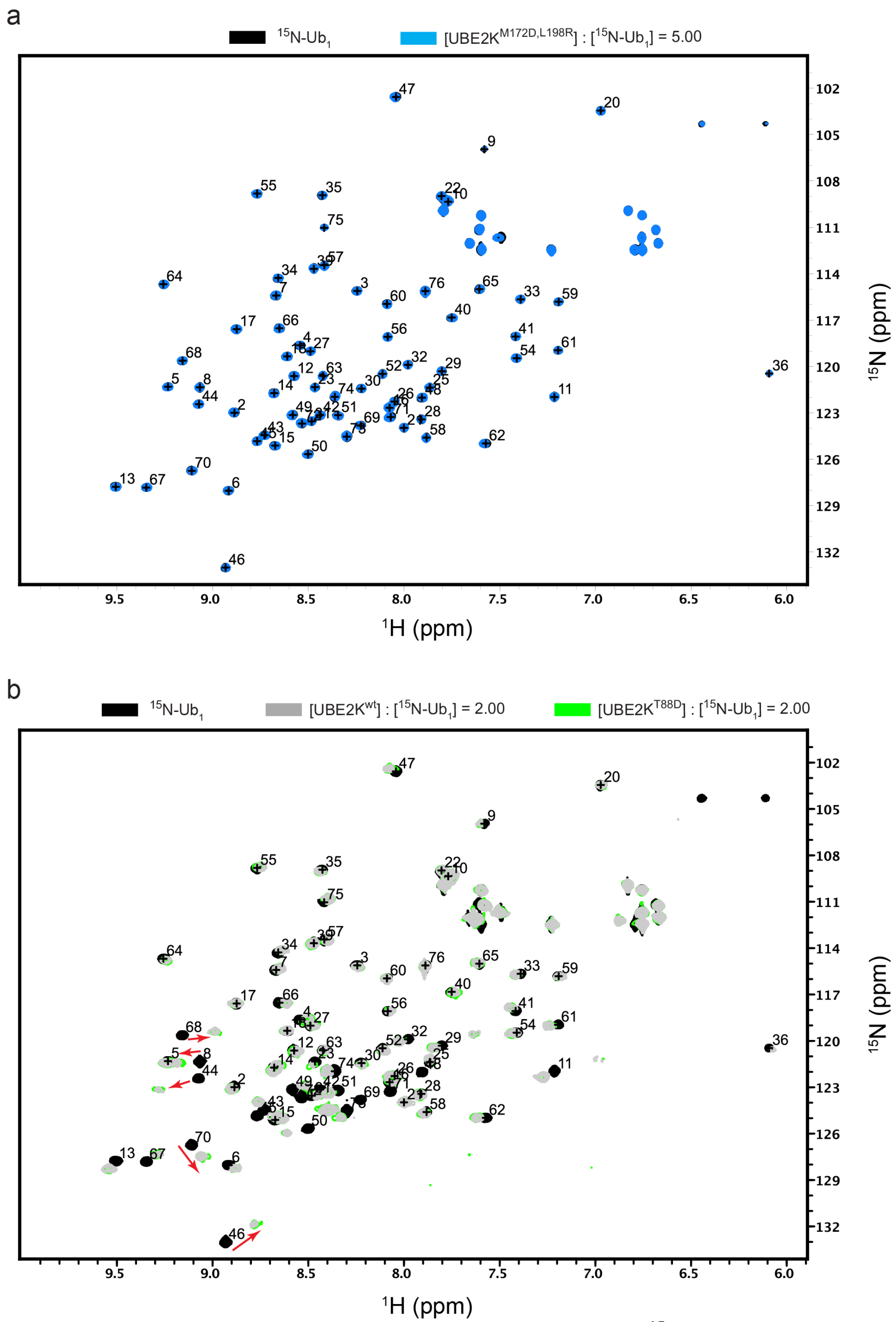

Supplementary Figure 3. NMR analysis of UBE2K variants and ^{15}N-Ub interaction.
(**a**) Overlay of ^{1}H-^{15}N HSQC spectrum of 200 µM ^{15}N-Ub (black) titrated with a five fold molar excess of UBE2K^{M172D,L198R} (blue), results in essentially no change in the spectrum. (**b**) Overlay of ^{1}H-^{15}N HSQC spectrum of 200 µM ^{15}N-Ub (black) titrated with a two fold molar excess of UBE2K^{WT} (grey) and UBE2K^{T88D} (green), demonstrates near identical binding to ^{15}N-Ub (red arrows indicate the trajectory for indicated Ub residues).

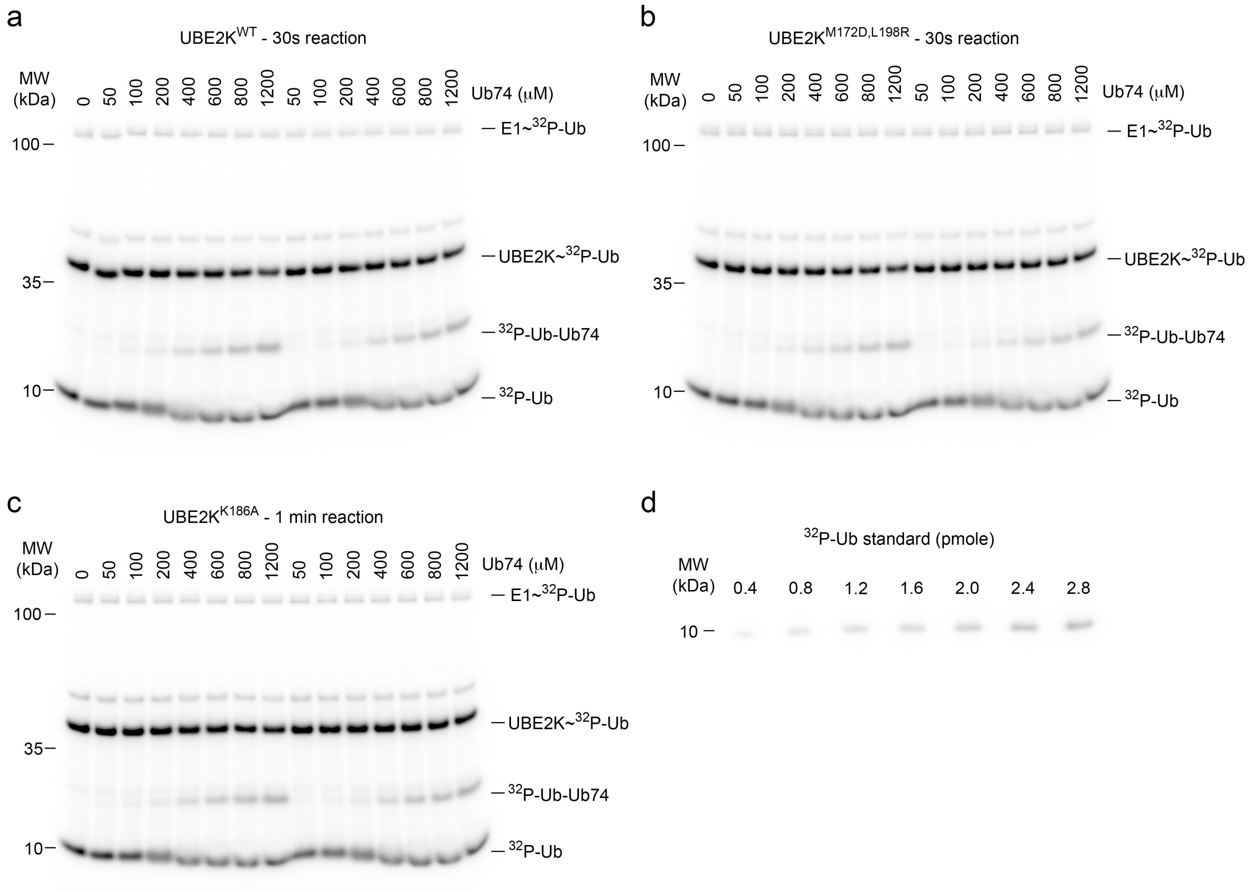

Supplementary Figure 4. Kinetic analysis of di-Ub formation catalyzed by UBE2K variants, related to Fig. 3f. Non-reduced autoradiograms showing the formation of ^{32}P-Ub-Ub74 over a range of Ub74 concentrations catalyzed by UBE2K^{WT} (**a**), UBE2K^{M172D,L198R} (**b**), and UBE2K^{L186A} (**c**). UBE2K variants are pre-charged with ^{32}P-Ub^{K48R}, added to varying Ub74 concentrations and stopped at indicated time as described in the Methods. Under this condition, less than 15% of UBE2K~^{32}P-Ub was used at all Ub74 concentrations and hence the reactions represented initial rates. All experiments were performed in duplicate. (**d**) Autoradiogram showing known amounts of ^{32}P-Ub used to generate a standard curve for quantification. All gels were exposed together to a phosphorimager screen and scanned with a Typhoon ™ PLA 7000. Unprocessed scan from Typhoon ™ PLA 7000 is shown in **Supplementary Figure 7**.

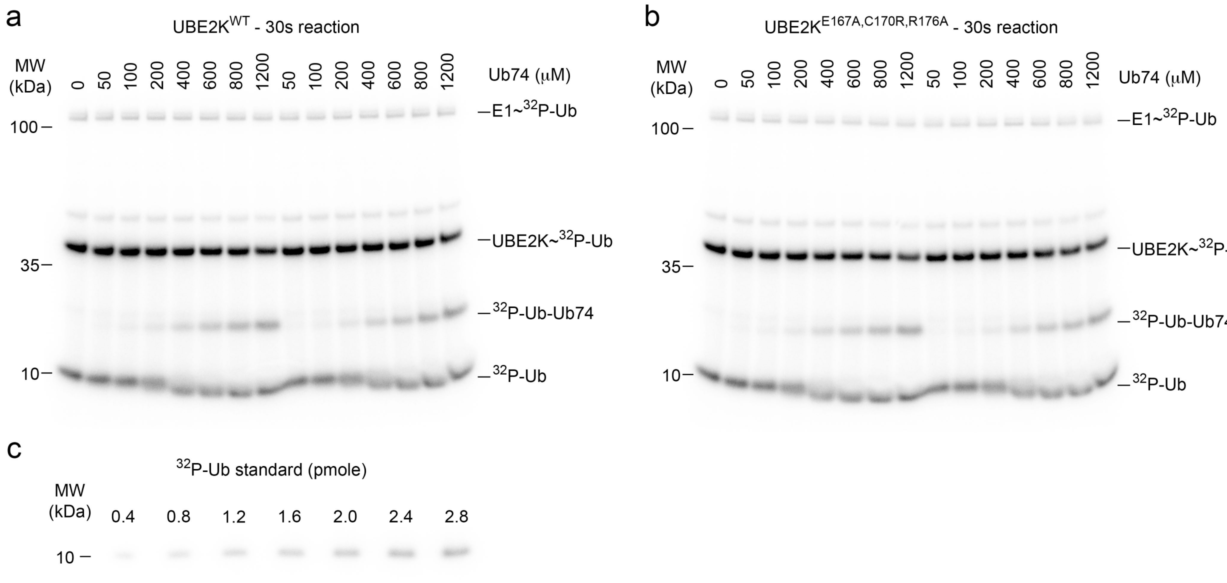

Supplementary Figure 5. Kinetic analysis of di-Ub formation catalyzed by UBE2K^{E167A,C170R,R176A}, related to Extended data Fig. 7h. Non-reduced autoradiograms showing the formation of ^{32}P-Ub-Ub74 over a range of Ub74 concentrations catalyzed by UBE2K^{WT} (**a**) and UBE2K^{E167A,C170R,R176A} (**b**). All experiments were performed in duplicate. (**c**) Autoradiogram showing known amounts of ^{32}P-Ub used to generate a standard curve for quantification. This is the same ^{32}P-Ub standard gel used in **Supplementary Fig. 4d** that was exposed together with gels from this set of experiments to a phosphorimager screen and scanned with a Typhoon ™ PLA 7000 for quantification. Unprocessed scan from Typhoon ™ PLA 7000 is shown in **Supplementary Figure 8**.

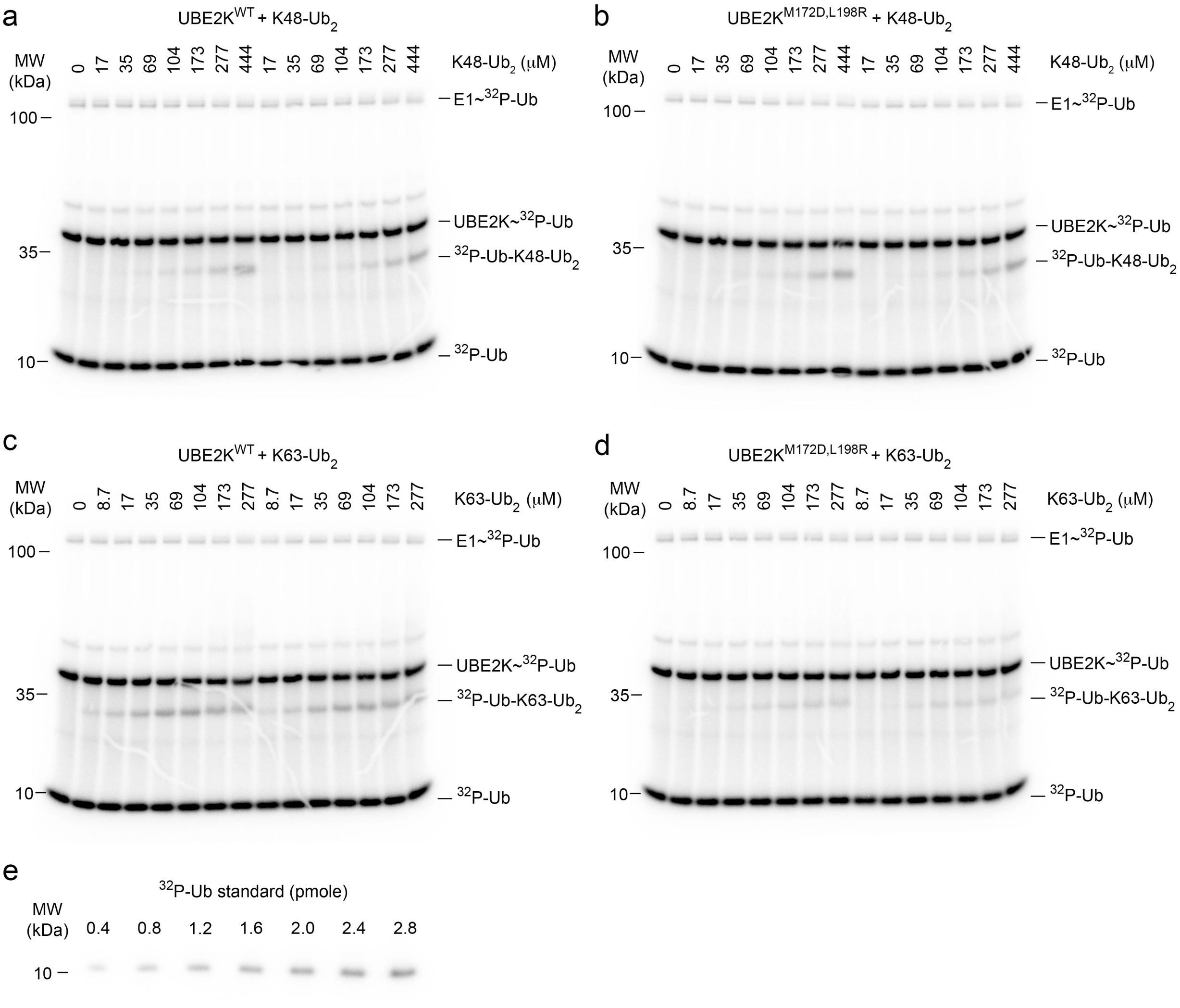

Supplementary Figure 6. Kinetic analysis of tri-Ub formation with K48-Ub_2 and K63-Ub_2 catalyzed by UBE2K, related to Fig. 6c,e. Non-reduced autoradiograms showing the formation of ^{32}P-Ub-K48-Ub_2 over a range of K48-Ub_2 concentrations catalyzed by $UBE2K^{WT}$ (**a**) and $UBE2K^{M172D,L198R}$ (**b**). Non-reduced autoradiograms showing the formation of ^{32}P-Ub-K63-Ub_2 over a range of K63-Ub_2 concentrations catalyzed by $UBE2K^{WT}$ (**c**) and $UBE2K^{M172D,L198R}$ (**d**). For **a–d**, all experiments were performed in duplicate. (**e**) Autoradiogram showing known amounts of ^{32}P-Ub used to generate a standard curve for quantification. This is the same ^{32}P-Ub standard gel used in **Supplementary Fig. 4d** that was exposed together with gels from this set of experiments to a phosphorimager screen and scanned with a Typhoon ™ PLA 7000 for quantification. Unprocessed scan from Typhoon ™ PLA 7000 is shown in **Supplementary Figure 9**.

Sup. Fig. 4a

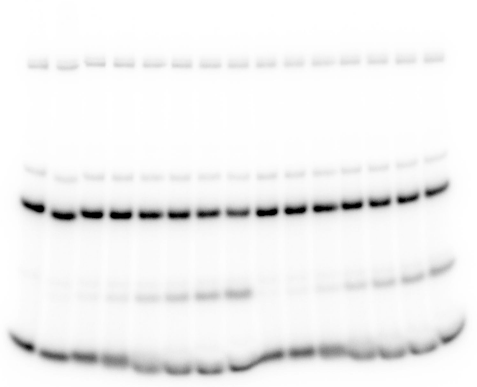

Sup. Fig. 4b

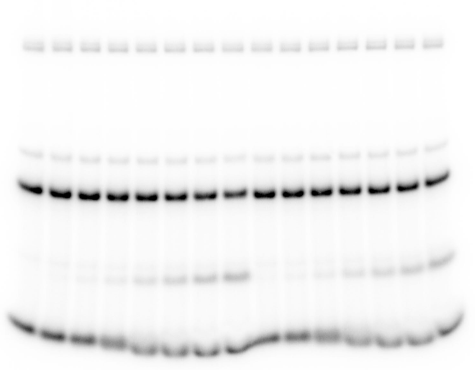

Sup. Fig. 4c

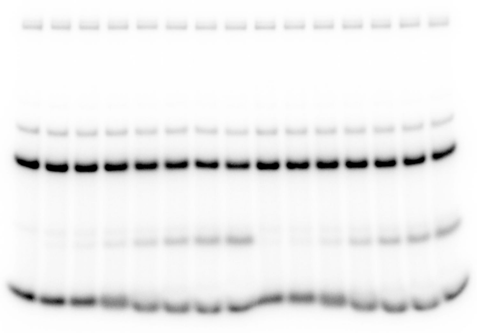

Sup. Fig. 4d

Supplementary Figure 7. Unprocessed autoradiograms for **Supplementary Figure 4**. All gels were exposed together to a phosphorimager screen and scanned with a Typhoon ™ PLA 7000.

Sup. Fig. 5a

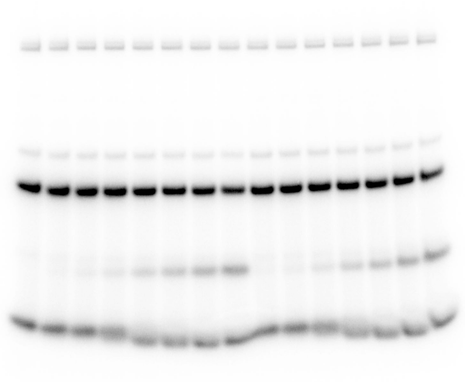

Sup. Fig. 5b

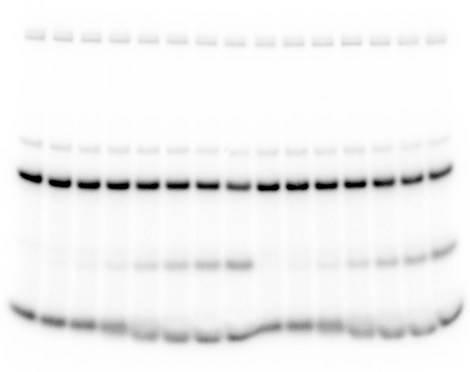

Sup. Fig. 5c

Supplementary Figure 8. Unprocessed autoradiograms for **Supplementary Figure 5**. All gels were exposed together to a phosphorimager screen and scanned with a Typhoon ™ PLA 7000.

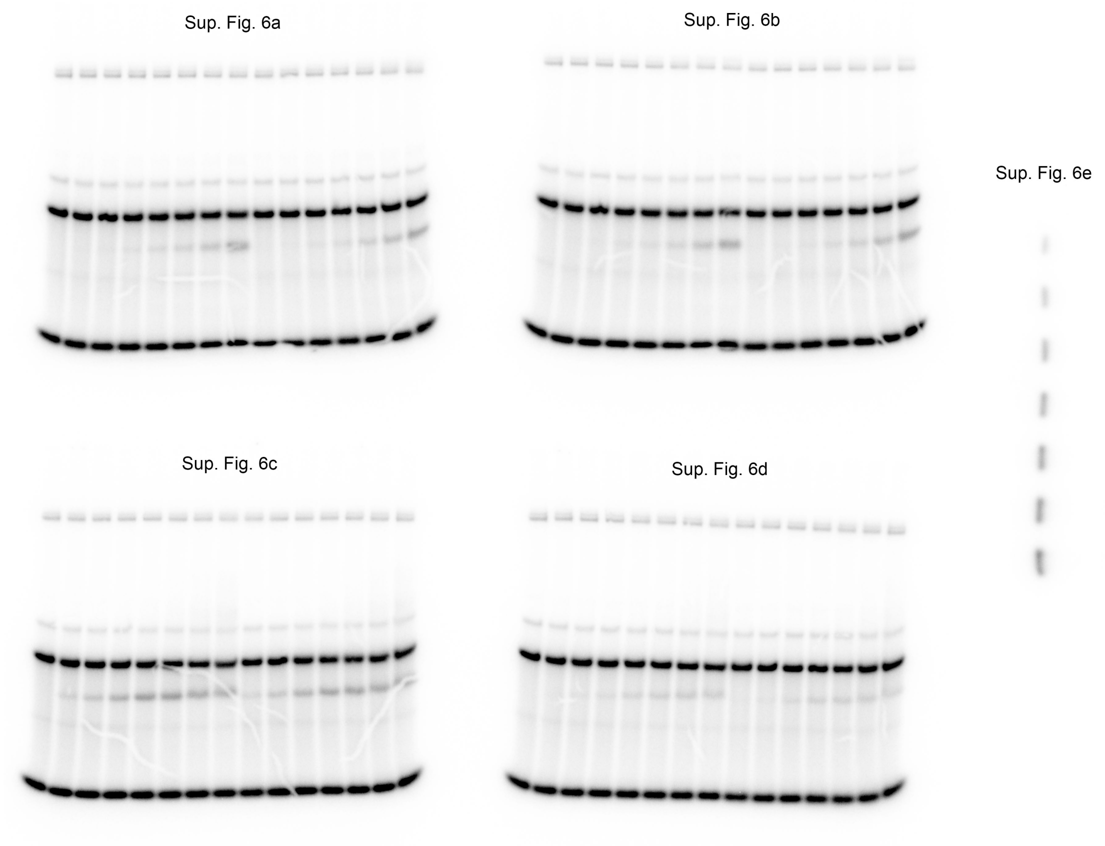

Supplementary Figure 9. Unprocessed autoradiograms for **Supplementary Figure 6**. All gels were exposed together to a phosphorimager screen and scanned with a Typhoon ™ PLA 7000.

Supplementary Table 1: Data collection and refinement statistics.

	Ub–^{C92K}UBE2K$^{D124C–K48C}$Ub–^{48}Ub/RNF38
**Data collection**	
Space group	P 31 2 1
Cell dimensions	
a, *b*, *c* (Å)	85.83, 85.83, 167.35
α, β, γ (°)	90, 90, 120
Resolution (Å)	74.34 – 2.40 (2.49 -2.40) *
R_{merge}	0.059 (0.97)
$I / \sigma I$	19.17 (0.72)
Completeness (%)	99.96 (99.93)
Redundancy	8.6 (8.7)
**Refinement**	
Resolution (Å)	74.34 – 2.40
No. reflections / test set	28632 / 1412
R_{work} / R_{free}	0.188 / 0.236
No. atoms	
Protein	3671
Ligand/ion	18
Water	50
B-factors	
Protein	94.81
Ligand/ion	112.88
Water	75.34
R.m.s. deviations	
Bond lengths (Å)	0.009
Bond angles (°)	1.11

*Values in parentheses are for highest-resolution shell.
