# Supplementary material for: Structure of UBE2K–Ub/E3/polyUb reveals mechanisms of K48-linked Ub chain extension
Source: Nat Chem Biol. 2022 Jan 13;18(4):422–31. doi: 10.1038/s41589-021-00952-x (PMC8964413; doi:10.1038/s41589-021-00952-x)
Supplement: Supplementary file 2 — Reporting Summary [file 41589_2021_952_MOESM2_ESM.pdf]

nature research

Corresponding author(s): Danny T. Huang

Last updated by author(s): Nov 8, 2021

Reporting Summary

Nature Research wishes to improve the reproducibility of the work that we publish. This form provides structure for consistency and transparency in reporting. For further information on Nature Research policies, see our Editorial Policies and the Editorial Policy Checklist.

Statistics

For all statistical analyses, confirm that the following items are present in the figure legend, table legend, main text, or Methods section.

n/a	Confirmed	
☐	☒	The exact sample size (n) for each experimental group/condition, given as a discrete number and unit of measurement
☐	☒	A statement on whether measurements were taken from distinct samples or whether the same sample was measured repeatedly
☒	☐	The statistical test(s) used AND whether they are one- or two-sided<br>*Only common tests should be described solely by name; describe more complex techniques in the Methods section.*
☒	☐	A description of all covariates tested
☒	☐	A description of any assumptions or corrections, such as tests of normality and adjustment for multiple comparisons
☐	☒	A full description of the statistical parameters including central tendency (e.g. means) or other basic estimates (e.g. regression coefficient) AND variation (e.g. standard deviation) or associated estimates of uncertainty (e.g. confidence intervals)
☒	☐	For null hypothesis testing, the test statistic (e.g. F, t, r) with confidence intervals, effect sizes, degrees of freedom and P value noted<br>*Give P values as exact values whenever suitable.*
☒	☐	For Bayesian analysis, information on the choice of priors and Markov chain Monte Carlo settings
☒	☐	For hierarchical and complex designs, identification of the appropriate level for tests and full reporting of outcomes
☒	☐	Estimates of effect sizes (e.g. Cohen's d, Pearson's r), indicating how they were calculated

Our web collection on statistics for biologists contains articles on many of the points above.

Software and code

Policy information about availability of computer code

Data collection	X-ray data were collected at beamline I04-1 at Diamond Light Source (UK). NMR data were acquired on a Bruker Avance III 600 MHz spectrometer with a cryogenic triple resonance inverse (TCI) probe.
Data analysis	X-ray datasets were processed with DIALS and the CCP4 program suite. Initial phases were obtained by molecular replacement with PHASER using structures of UBE2K (PDB: 1YLA), Ub (PDB:1UBQ) and RNF38 RING domain (PDB:4V3L) as search models. Refinement was performed using COOT and PHENIX. NMR data were processed with Bruker TopSpin version 3.5 patch level 7 and analyzed using CARA and NMRFAM-SPARKY. Licor ImageStudio v5 and the Fiji distribution of ImageJ were used for In-gel and Western blot quantification. Prism (Graphpad) was used to generate plots. Pymol was used to generate structural figures. 32P Kinetic experiments were analyzed with Typhoon ™ PLA 7000 (GE Healthcare) and quantified using ImageQuant (GE Healthcare). Modeling of the native structure and energy minimization was done using GalaxyRefineComplex.

For manuscripts utilizing custom algorithms or software that are central to the research but not yet described in published literature, software must be made available to editors and reviewers. We strongly encourage code deposition in a community repository (e.g. GitHub). See the Nature Research guidelines for submitting code & software for further information.

Data

Policy information about availability of data

All manuscripts must include a data availability statement. This statement should provide the following information, where applicable:

- Accession codes, unique identifiers, or web links for publicly available datasets
- A list of figures that have associated raw data
- A description of any restrictions on data availability

Coordinates and structure factors have been deposited with the protein data bank under accession code 7OJX. All data and DNA constructs are available from the

corresponding author.

Field-specific reporting

Please select the one below that is the best fit for your research. If you are not sure, read the appropriate sections before making your selection.

☒ Life sciences ☐ Behavioural & social sciences ☐ Ecological, evolutionary & environmental sciences

For a reference copy of the document with all sections, see nature.com/documents/nr-reporting-summary-flat.pdf

Life sciences study design

All studies must disclose on these points even when the disclosure is negative.

Sample size	The sample size varied between 2-4 for in vitro experiments. This sample size was used to verify experimental reproducibility and to obtain mean +/- SD where applicable.
Data exclusions	No data were excluded.
Replication	Three independent reactions were performed for quantification of activity at a single reaction time point. For kinetic and SPR analyses, two independent reactions were performed. All replicates were successful and none were omitted.
Randomization	There was no randomization in this study. This was not applicable in our study.
Blinding	There was no blinding in this study. This was not applicable in our study.

Reporting for specific materials, systems and methods

We require information from authors about some types of materials, experimental systems and methods used in many studies. Here, indicate whether each material, system or method listed is relevant to your study. If you are not sure if a list item applies to your research, read the appropriate section before selecting a response.

Materials & experimental systems

n/a	Involved in the study	
☐	☒	Antibodies
☒	☐	Eukaryotic cell lines
☒	☐	Palaeontology and archaeology
☒	☐	Animals and other organisms
☒	☐	Human research participants
☒	☐	Clinical data
☒	☐	Dual use research of concern

Methods

n/a	Involved in the study	
☒	☐	ChIP-seq
☒	☐	Flow cytometry
☒	☐	MRI-based neuroimaging

Antibodies

Antibodies used	Primary antibodies were mouse anti-ubiquitin (Santa Cruz , cat. no sc-8017, clone no. P4D1, Lot no. F0718, 1:5,000 for Western blot), rabbit anti-K48 linkage specific polyubiquitin (Cell Signaling Technology, cat. no 8081S, clone no. D9D5, Lot no. 4, 1:5,000 for Western blot), and mouse anti-Strep Tag-II (Merk , cat. no 71590, Lot no. 2731204, 1:2,000 for Western blot).The secondary antibodies used were goat anti-mouse IRDye 800CW (LI-COR Biosciences, cat no. 925-32210, Lot no. C61012-06, 1:15000 for Western blot) and goat anti-rabbit IRDye 800CW (LI-COR Biosciences, cat no. 926-3221, Lot no. C91211-03, 1:15000 for Western blot)
Validation	Goat anti-mouse IRDye 800CW (https://www.licor.com/bio/reagents/irdye-800cw-goat-anti-mouse-igg-secondary-antibody)<br>Goat anti-rabbit IRDye 800CW (https://www.licor.com/bio/reagents/irdye-800cw-goat-anti-rabbit-igg-secondary-antibody)<br>rabbit anti-K48 linkage specific polyubiquitin (https://www.cellsignal.co.uk/products/primary-antibodies/k48-linkage-specific-polyubiquitin-d9d5-rabbit-mab/8081)<br>mouse anti-Ub (https://www.scbt.com/p/ubiquitin-antibody-p4d1)<br>mouse anti-Strep Tag-II (https://www.merckmillipore.com/GB/en/product/StrepTag-II-Monoclonal-Antibody,EMD_BIO-71590)
