# Supplementary figures and images for: Structure of UBE2K–Ub/E3/polyUb reveals mechanisms of K48-linked Ub chain extension
Source: Nat Chem Biol. 2022 Jan 13;18(4):422–31. doi: 10.1038/s41589-021-00952-x (PMC8964413; doi:10.1038/s41589-021-00952-x)

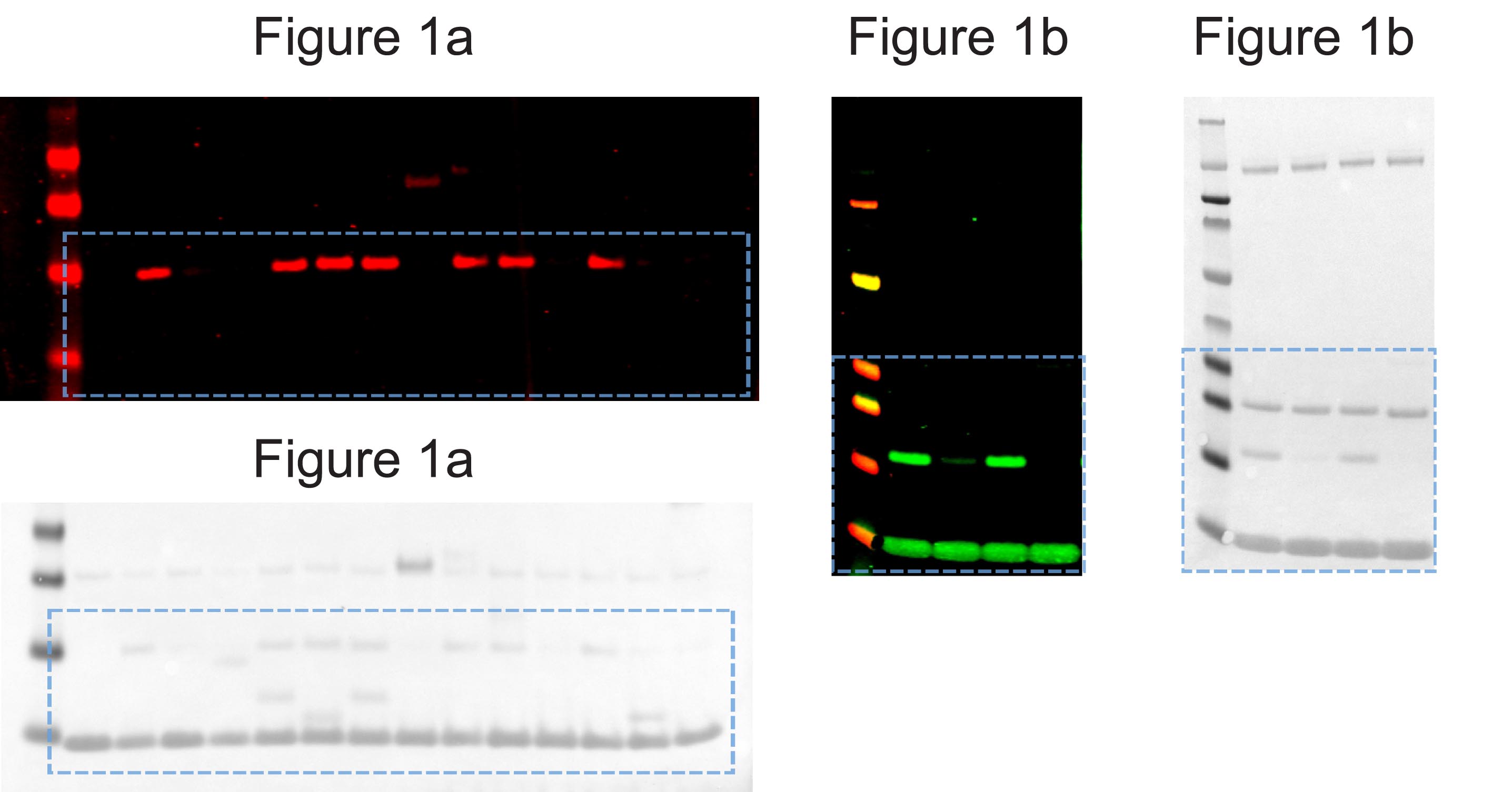

Supplement: Source Data Fig. 1 — Unprocessed gels for Fig. 1. [file 41589_2021_952_MOESM3_ESM.jpg]

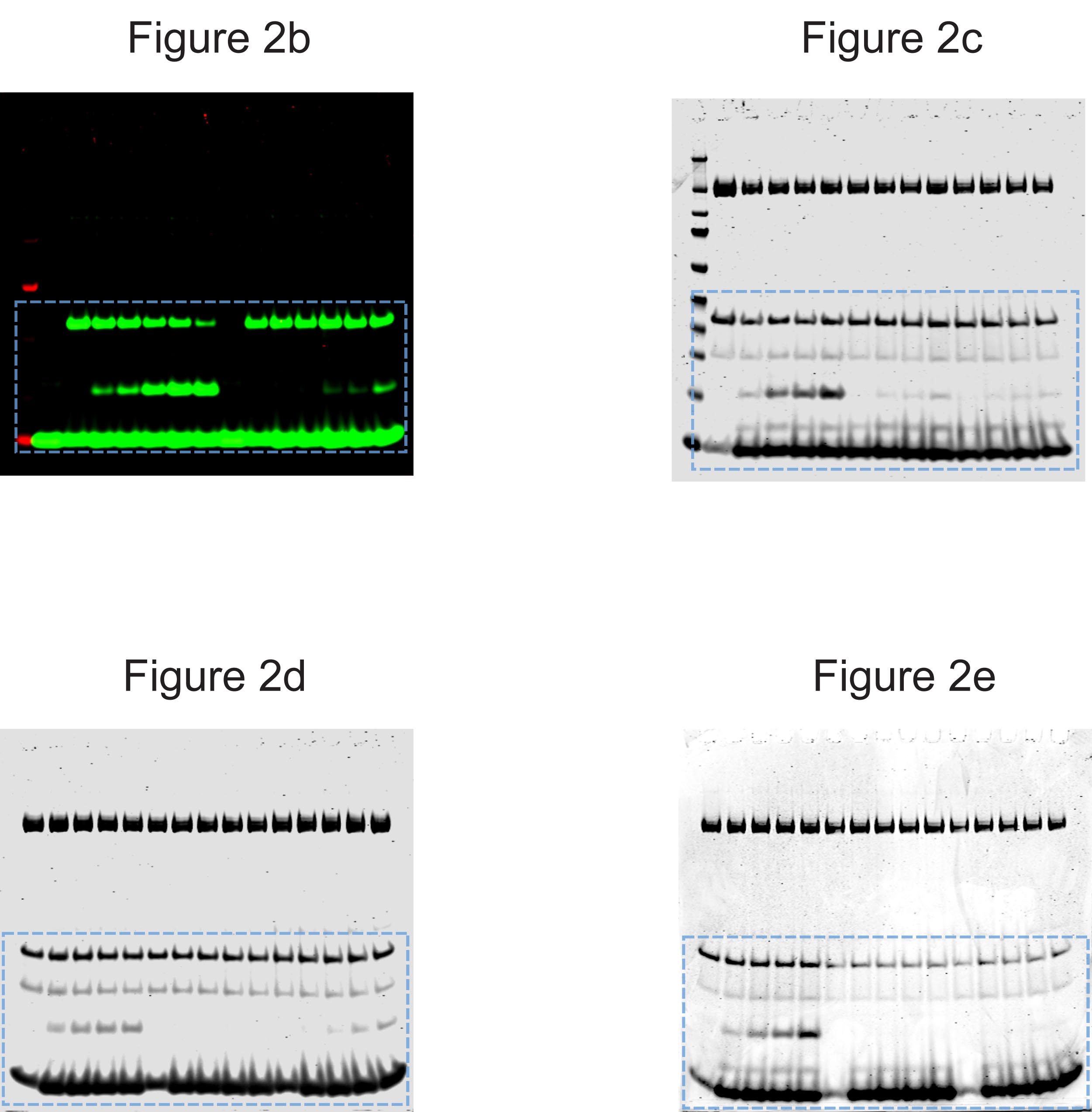

Supplement: Source Data Fig. 2 — Unprocessed gels for Fig. 2. [file 41589_2021_952_MOESM4_ESM.jpg]

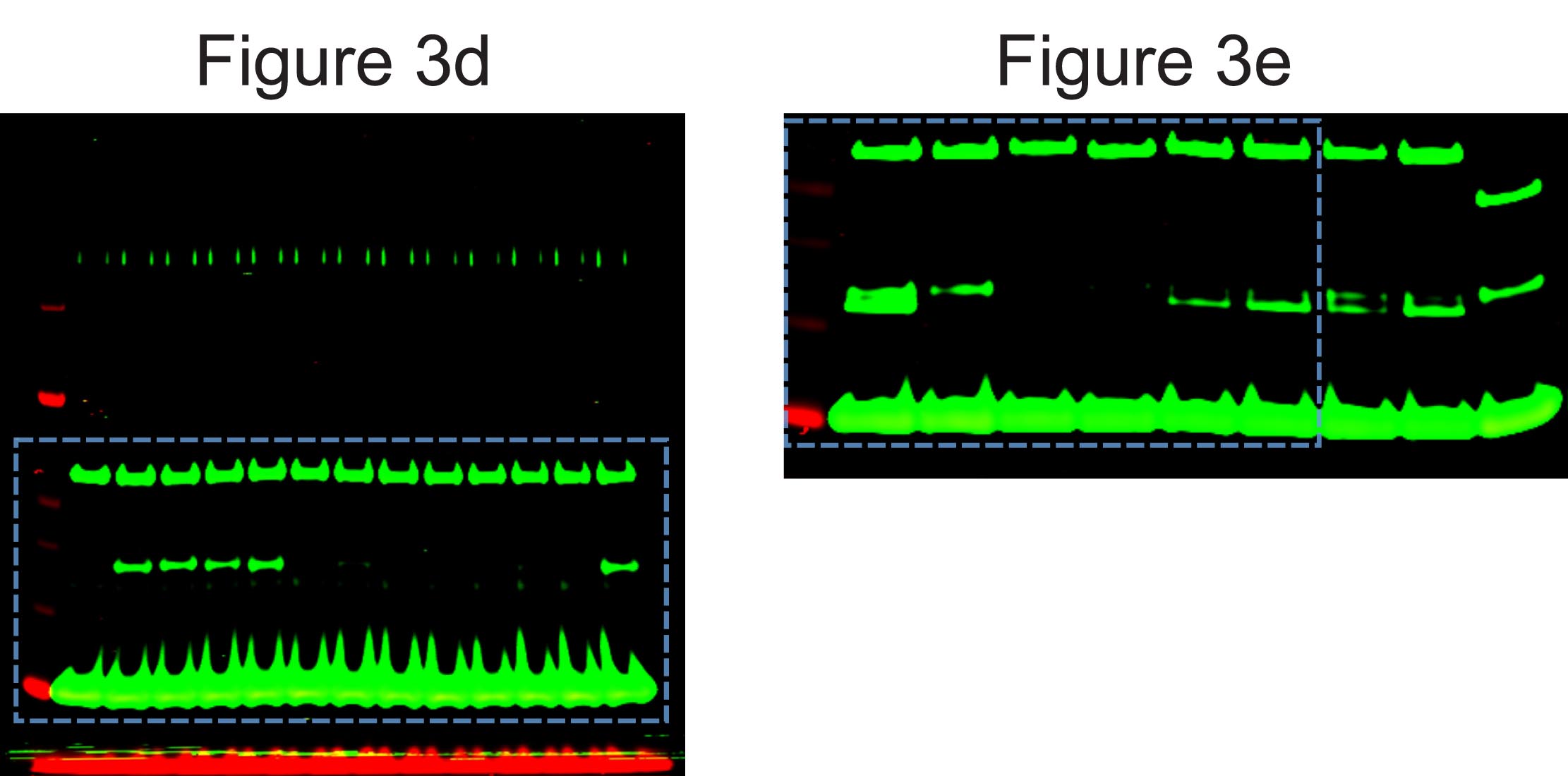

Supplement: Source Data Fig. 3 — Unprocessed gels for Fig. 3. [file 41589_2021_952_MOESM5_ESM.jpg]

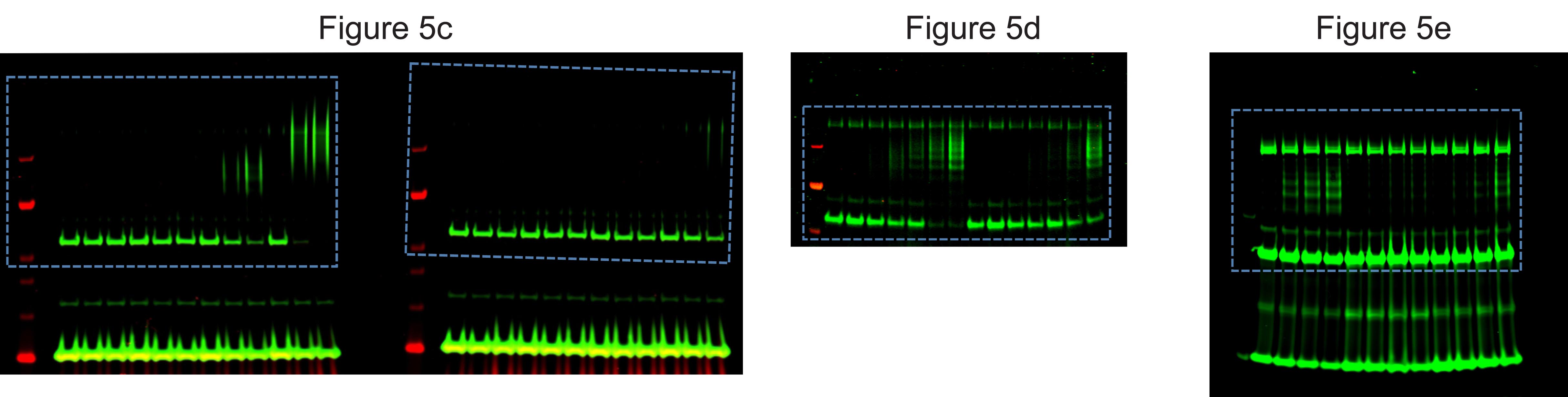

Supplement: Source Data Fig. 5 — Unprocessed gels for Fig. 5. [file 41589_2021_952_MOESM6_ESM.jpg]

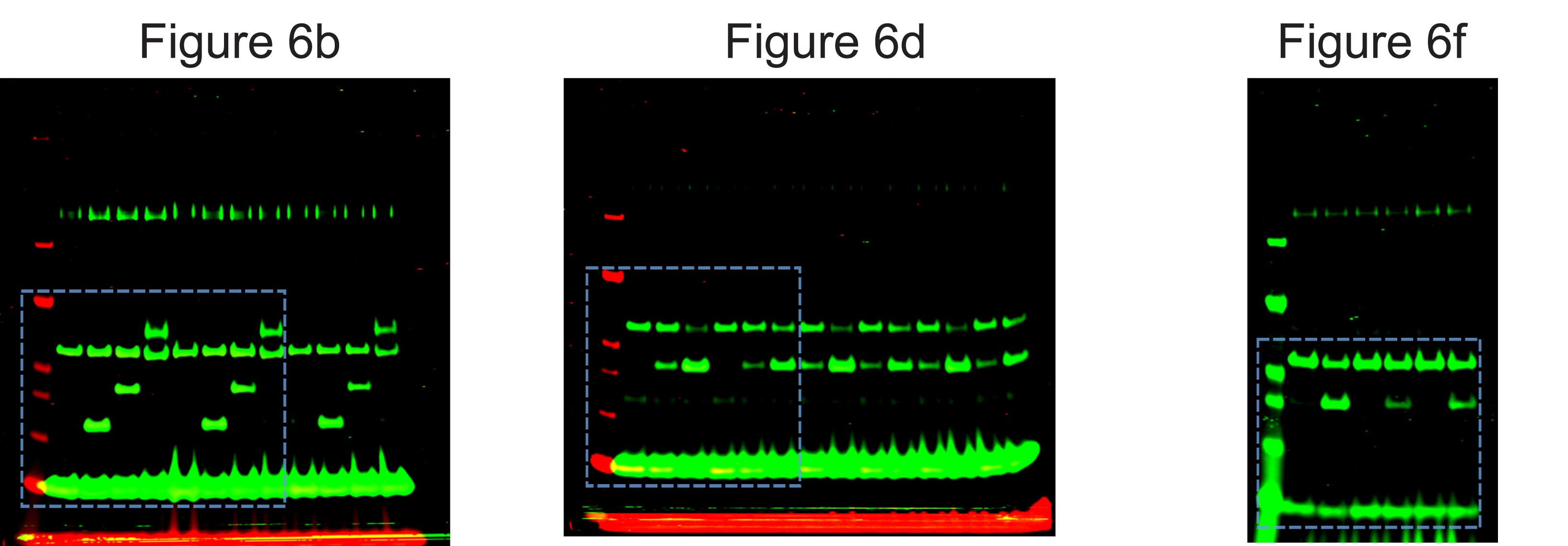

Supplement: Source Data Fig. 6 — Unprocessed gels for Fig. 6. [file 41589_2021_952_MOESM7_ESM.jpg]

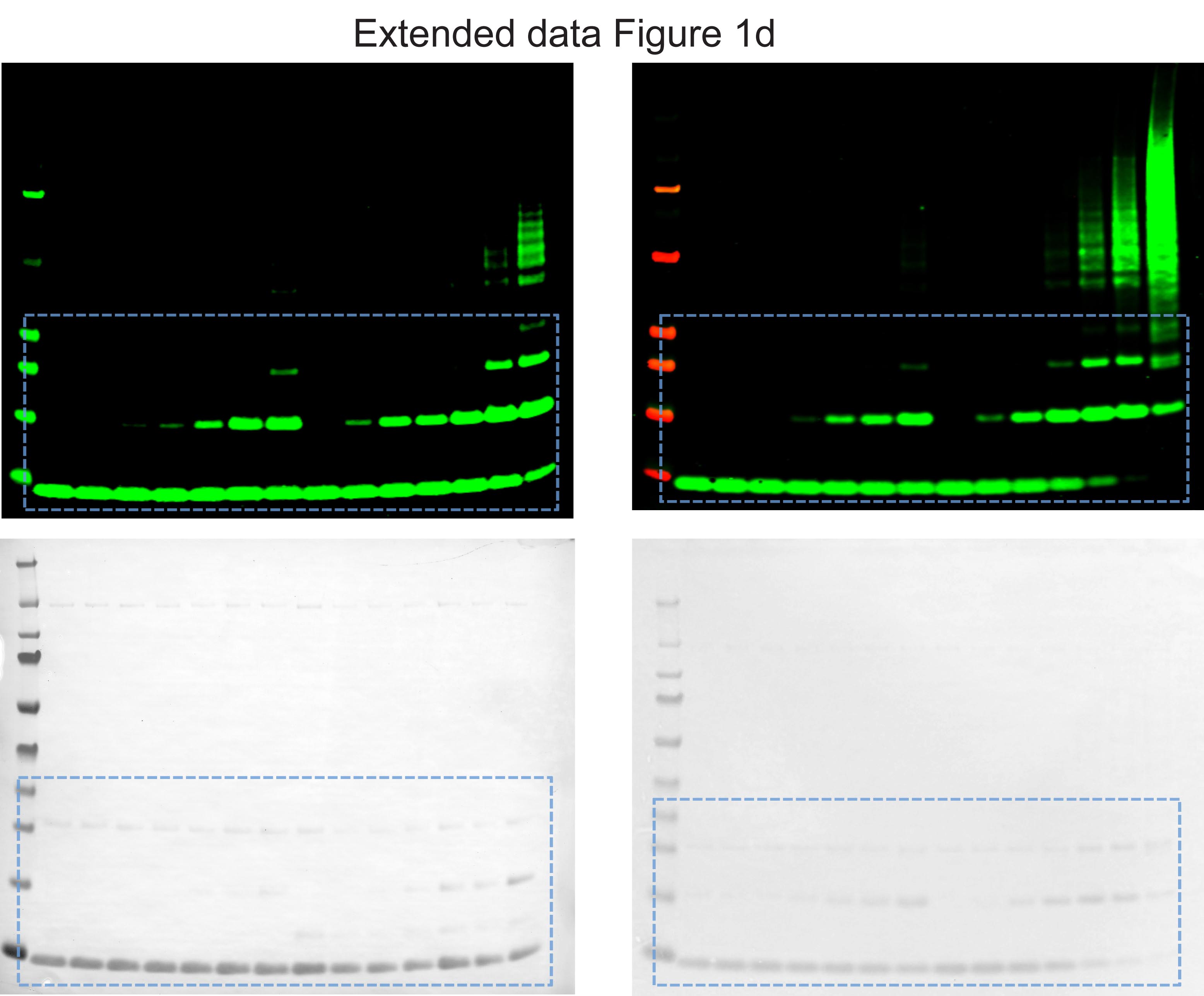

Supplement: Source Data Extended Data Fig. 1 — Unprocessed gels for Extended Data Fig. 1. [file 41589_2021_952_MOESM8_ESM.jpg]

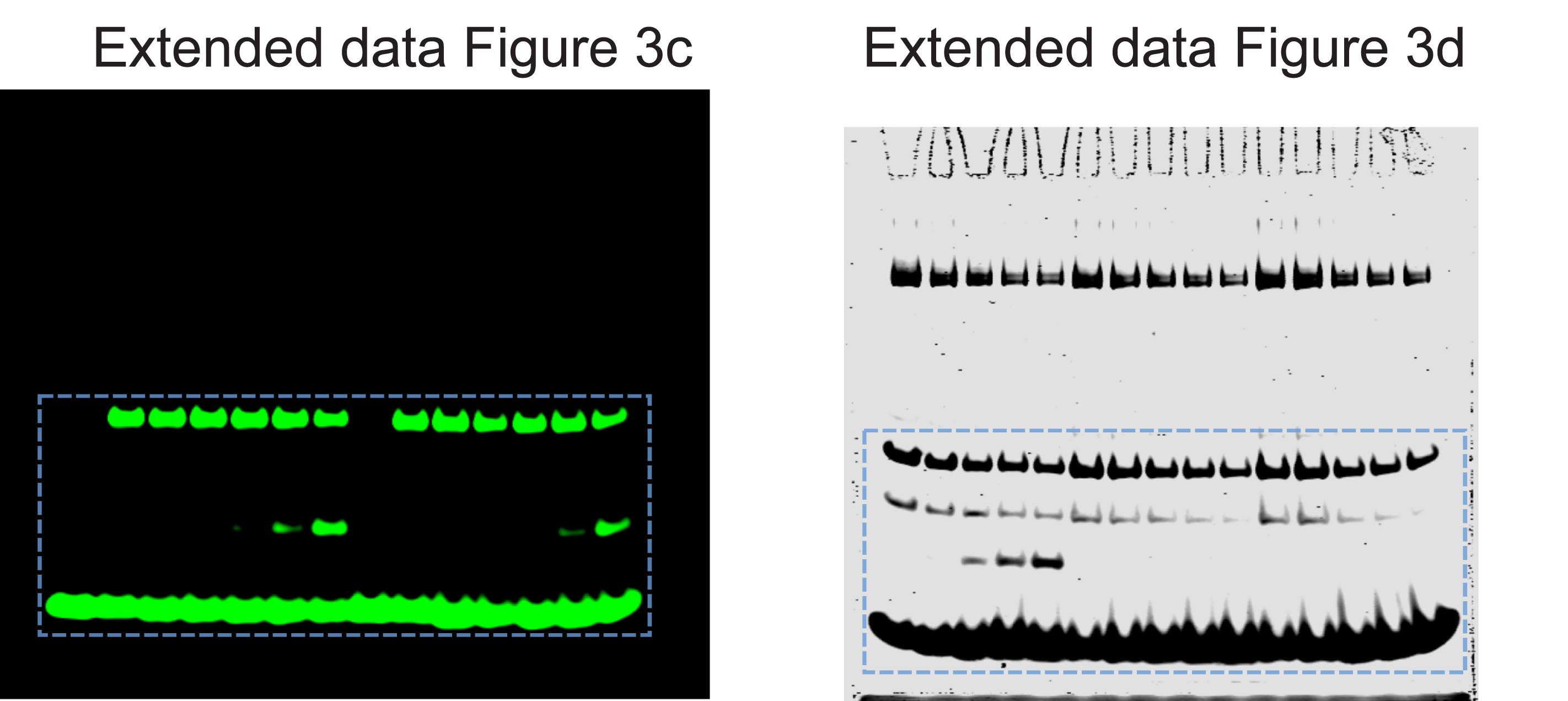

Supplement: Source Data Extended Data Fig. 3 — Unprocessed gels for Extended Data Fig. 3. [file 41589_2021_952_MOESM9_ESM.jpg]

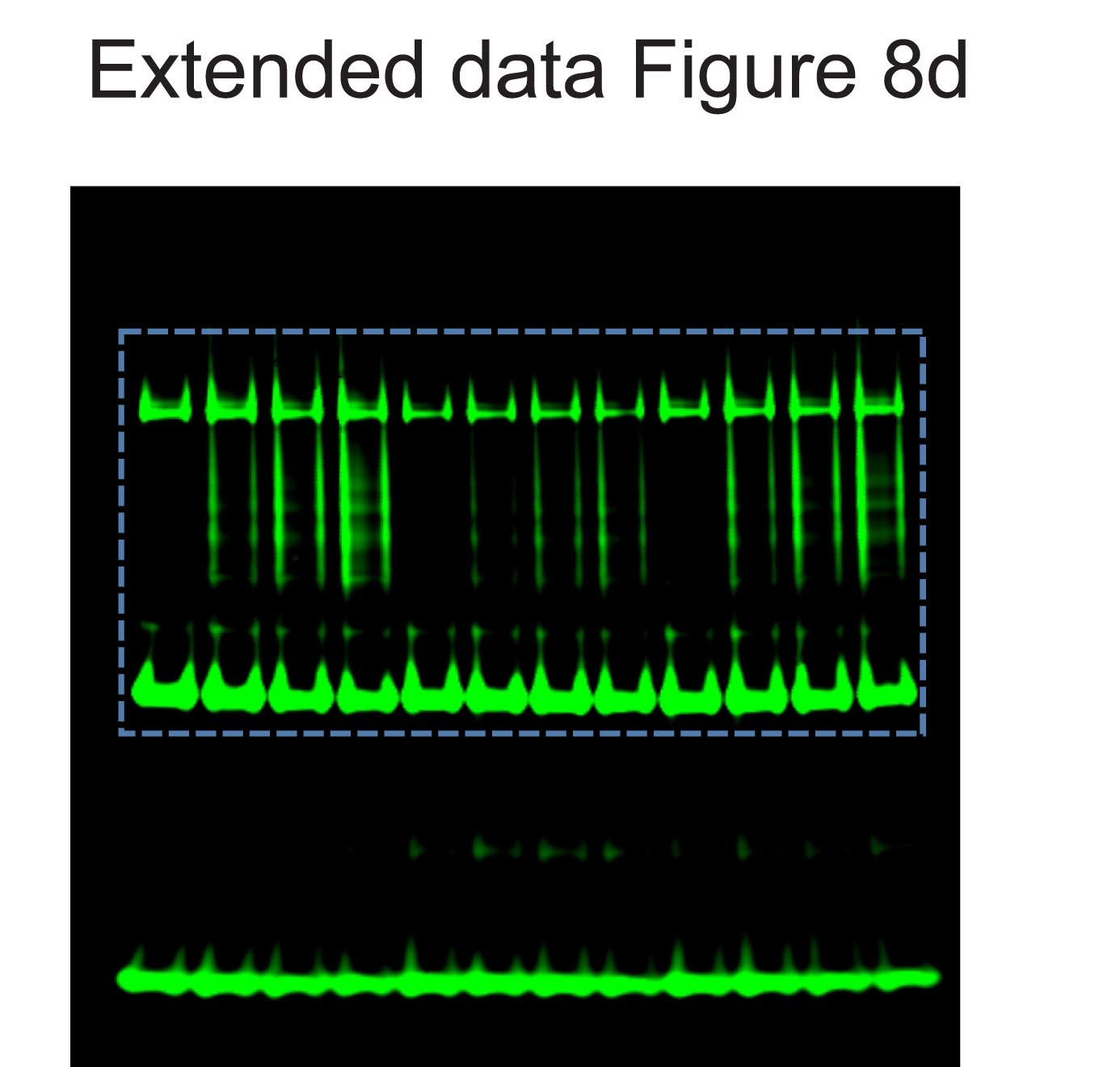

Supplement: Source Data Extended Data Fig. 8 — Unprocessed gels for Extended Data Fig. 8. [file 41589_2021_952_MOESM10_ESM.jpg]

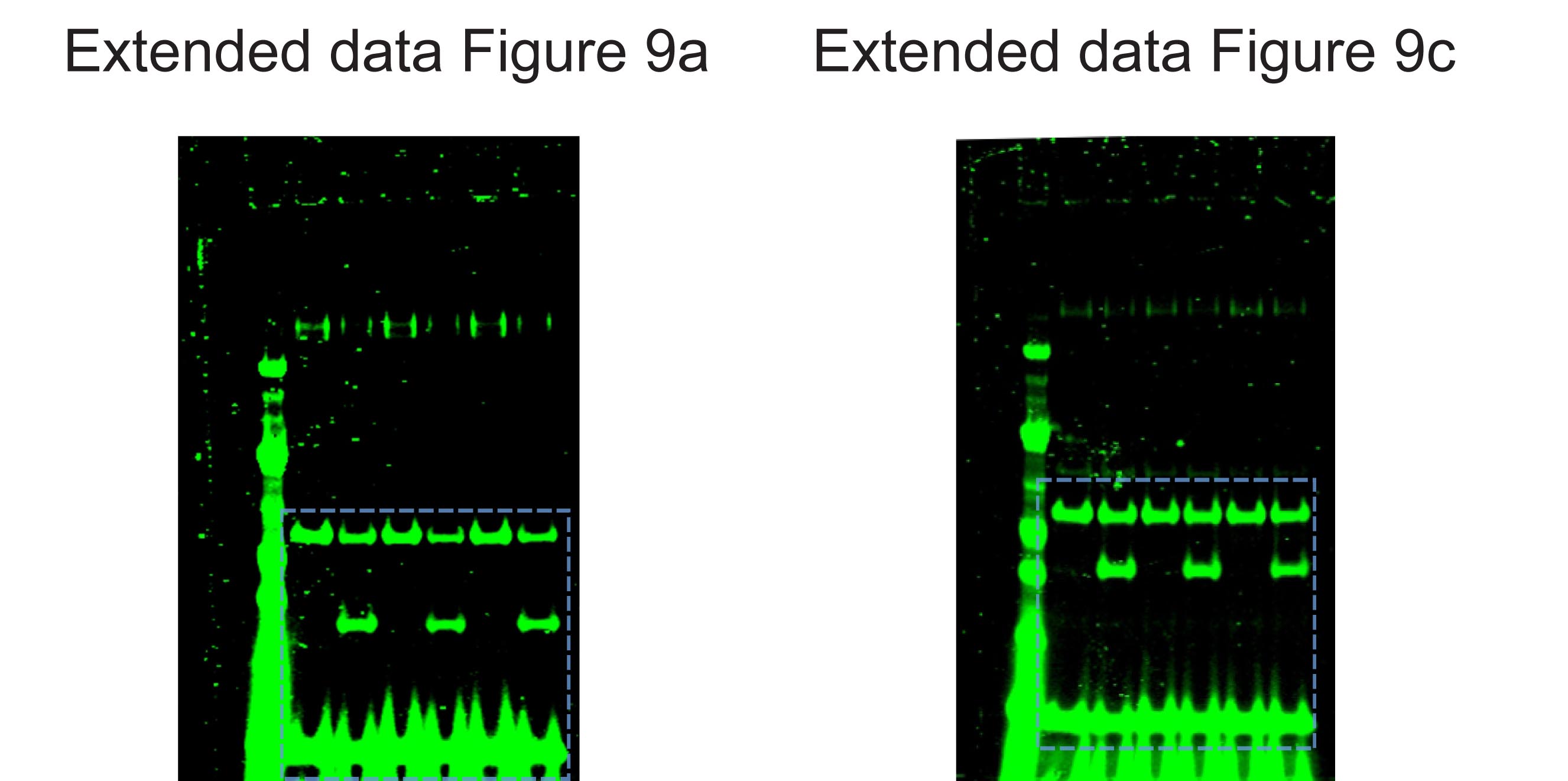

Supplement: Source Data Extended Data Fig. 9 — Unprocessed gels for Extended Data Fig. 9. [file 41589_2021_952_MOESM11_ESM.jpg]
